# 5-aminoimidazole-4-carboxamide ribonucleoside induces differentiation in a subset of primary acute myeloid leukemia blasts

**DOI:** 10.1186/s12885-020-07533-6

**Published:** 2020-11-11

**Authors:** Vilma Dembitz, Hrvoje Lalic, Ivan Kodvanj, Barbara Tomic, Josip Batinic, Klara Dubravcic, Drago Batinic, Antonio Bedalov, Dora Visnjic

**Affiliations:** 1 Croatian Institute for Brain Research, University of Zagreb School of Medicine, 10 000 Zagreb, Croatia; 2 Department of Physiology, University of Zagreb School of Medicine, Zagreb, Croatia; 3 Division of Hematology, Department of Internal Medicine, University Hospital Center Zagreb, Zagreb, Croatia; 4 Department of Laboratory Immunology, University Hospital Center Zagreb, Zagreb, Croatia; 5 Clinical Research Division, Fred Hutchinson Cancer Research Center, Seattle, WA USA

**Keywords:** AICAr, Acute myeloid leukemia, Differentiation, Brequinar, ATRA

## Abstract

**Background:**

All*-trans* retinoic acid (ATRA)-based treatment of acute promyelocytic leukemia (APL) is the most successful pharmacological treatment of acute myeloid leukemia (AML). Recent development of inhibitors of mutated isocitrate dehydrogenase and dihydroorotate dehydrogenase (DHODH) has revived interest in differentiation therapy of non-APL AML. Our previous studies demonstrated that 5-aminoimidazole-4-carboxamide ribonucleoside (AICAr) induced differentiation of monocytic cell lines by activating the ATR/Chk1 via pyrimidine depletion. In the present study, the effects of AICAr on the viability and differentiation of primary AML blasts isolated from bone marrow of patients with non-APL AML were tested and compared with the effects of DHODH inhibitor brequinar and ATRA.

**Methods:**

Bone marrow samples were obtained from 35 patients and leukemia blasts were cultured ex vivo. The cell viability was assessed by MTT assay and AML cell differentiation was determined by flow cytometry and morphological analyses. RNA sequencing and partial data analysis were conducted using ClusterProfiler package. Statistical analysis was performed using GraphPad Prism 6.0.

**Results:**

AICAr is capable of triggering differentiation in samples of bone marrow blasts cultured ex vivo that were resistant to ATRA. AICAr-induced differentiation correlates with proliferation and sensitivity to DHODH inhibition. RNA-seq data obtained in primary AML blasts confirmed that AICAr treatment induced downregulation of pyrimidine metabolism pathways together with an upregulation of gene set involved in hematopoietic cell lineage.

**Conclusion:**

AICAr induces differentiation in a subset of primary non-APL AML blasts, and these effects correlate with sensitivity to a well-known, potent DHODH inhibitor.

**Supplementary information:**

**Supplementary information** accompanies this paper at 10.1186/s12885-020-07533-6.

## Background

Acute myeloid leukemia (AML) is a severe hematological disorder that is characterized by the clonal expansion of myeloid blasts arrested at different stages of differentiation. AML is the most common acute type of leukemia in adults, with a median age at diagnosis of 68 years, the high risk of relapse and high mortality rate. The standard cytotoxic therapy consists of 7 days of cytarabine combined with 3 days of an anthracycline as a remission induction therapy, and several courses of high dose cytarabine or allogeneic hematopoietic stem cell transplantation as a consolidation therapy. With this therapy, long-term survival for patients with AML is achieved in only 35–45% of those younger than 60 years of age and 10–15% of those aged 60 years and older. In elderly patients for whom intensive therapy is not appropriate, treatment remains unsatisfactory and includes low-dose cytarabine or the hypomethylating agents with supportive care. Since 2017, several new drugs have been approved for the treatment of AML, including FLT3 inhibitors, the CD33-directed antibodies and inhibitors of mutated isocitrate dehydrogenase (IDH) [1, 2]. However, the most successful pharmacological treatment of AML is still *all-trans* retinoic acid (ATRA)-based differentiation therapy of acute promyelocytic leukemia (APL), a particular subtype of AML defined by the PML-RARA rearrangement. Once fatal disease, APL has been successfully treated for the last three decades with ATRA and chemotherapy with complete remission (CR) rates of 90 to 95%, and 85 to 90% rates of long-term survival. The clinical outcome of the disease has been further improved by the introduction of arsenic trioxide (ATO) into the treatment of refractory or relapsed APL, or as a first-line treatment in combination with ATRA [3].

The proposed mechanism of ATRA action involves binding of ATRA to the fusion protein promyelocytic leukemia (PML)/retinoic acid receptor α (RARα) that is encoded by a translocation involving *PML* gene on chromosome 15 and *RARA* gene on chromosome 17, and which is assumed to act as a co-repressor. Therefore, ATRA-based therapy is reserved for cells having PML-RARA fusion product, which account for only 10–15% of AML, and all other non-APL AML are treated with cytotoxic drugs. However, it should be noted that differentiative properties of ATRA were first described in HL-60 cell line [4] established from peripheral blood of a patient suffering from AML-M2 which actually lack the PML-RARA rearrangement, and several studies showed that ATRA may drive leukemic cells efficiently into differentiation and/or apoptosis in a subset of non-APL AML patients [5]. Recent findings that inhibitors of mutated isocitrate dehydrogenase (IDH) are associated with clinical and morphological signs of myeloid differentiation revived the interest in differentiation therapy of non-APL AML. Mutations in *IDH* genes are present in 18–22% of AML and lead to accumulation of the oncometabolite R-2-hydroxyglutarate (2-HG), which ultimately creates epigenetic alterations and differentiation arrest of AML blasts. IDH inhibitors restore normal enzyme activity, reduce 2-HG and induce differentiation [6, 7] being well-tolerated even in elderly patients with multiple comorbidities [8]. The IDH2 inhibitor enasidenib and the IDH1 inhibitor ivosidenib received FDA approval in 2017 and 2018 for selected patients with *IDH2*- and *IDH1*-mutated relapsed and refractory (R/R) AML, respectively.

The success of IDH inhibitors invigorated interest in the role of metabolism in differentiation so that several new enzymes have been reported as possible targets for differentiation of AML, including methylenetetrahydrofolate dehydrogenase-cyclohydrolase 2 (MTHFD2) [9], dihydroorotate dehydrogenase (DHODH) [10] and lysine-specific demethylases [11]. Our previous studies demonstrated that 5-aminoimidazole-4-carboxamide ribonucleoside (AICAr), a widely used agonist of AMP-activated kinase (AMPK), induces differentiation of monocytic leukemia cell lines in an AMPK-independent manner [12]. Ribotide AICAR was shown to interfere with pyrimidine synthesis by inhibiting UMP-synthase, which is a step downstream of DHODH; AICAr and the DHODH inhibitor brequinar had similar effects on differentiation and S-phase arrest, and the effects of both drugs depended on the activation of the DNA damage response ATR/Chk1 pathway [13]. In last couple of years, multiple agents have been identified as potent inhibitors of DHODH that trigger some differentiation of AML cell lines [14–19], patient AML cells ex vivo [19] or patient-derived xenografts in vivo [18, 19], and four clinical trials are underway in order to establish their safety and efficiency in AML patients [20].

The present study is a continuation of our previous work demonstrating the mechanism of AICAr-mediated differentiation [12, 13] that is undertaken in order to determine whether data obtained in AML cell lines could be translated to primary AML samples. Results of the study show that AICAr is capable of triggering differentiation in samples of bone marrow blasts cultured ex vivo that were resistant to ATRA. AICAr-induced differentiation correlates with proliferation and sensitivity to DHODH inhibition.

## Methods

### Reagents

Reagents used are listed in Supplementary Table 1.

### Cell culture

Bone marrow samples were obtained from 35 patients with non-APL AML at the time of diagnosis. None of the patients have received any treatment before specimens were collected. All patients provided written informed consent in accordance with the Declaration of Helsinki and the study was approved by the Institutional Review Board of the University of Zagreb School of Medicine (380–59–10,106-17-100/94) and University Hospital Center Zagreb (02/21 AG). In routine diagnostic procedures, all patients were tested for cytogenetic abnormalities and *FLT3* mutations. Patients with normal karyotypes were additionally tested for *NPM1* mutations. Depending on the FAB subtype, further analyses of BCR-ABL, AML1-ETO, PML-RARA, CBFB/MYH11 and MLL-AF4/AF9 were performed.

Mononuclear cells from bone marrow aspirates were isolated by density gradient centrifugation with NycoPrep™ 1.077, further purified by overnight adherence to plastic and cryopreserved in liquid nitrogen. Thawed cells were cultured at starting concentration 0.4 × 10^6^/mL in RPMI 1640 containing 10% FBS, 2 mM L-glutamine, 50 U/mL penicillin and 50 μg/mL streptomycin supplemented with 50 ng/mL interleukin-3 (IL-3), interleukin-6 (IL-6), FLT3 ligand (FLT3L) and stem cell factor (SCF) as previously described [21] and treated with agents tested. Only 16 samples with cell concentration higher than 0.2 × 10^6^/mL in control conditions after 72 h culture were considered viable and included in further analysis. AML patients’ characteristics are presented in Supplementary Table 2.

U937 cell line (ECACC number 85011440) was purchased from the European Collection of Authenticated Cell Cultures (Porton, Salisbury, UK). Cell line was expanded and frozen at early passages and used for the experiments within 10 weeks after being thawed from frozen stocks. The cells were maintained in RPMI 1640 medium containing 10% FBS, 2 mM L-glutamine, 50 U/mL penicillin and 50 μg/mL streptomycin at 37 °C in a humidified atmosphere containing 5% CO_2_. For the experiments, cells were harvested, resuspended in fresh medium, seeded at a concentration of 0.2 × 10^6^/mL in 6-well plates and treated with or without 0.2 mM AICAr for 24 h.

### Cell viability

The total cell number and viability were assessed by counting on hemocytometer using trypan blue exclusion and by 3-(4,5-dimethylthiazol-2-yl)-2,5-diphenyltetrazolium bromide (MTT) assay. For MTT assay, cells were grown in triplicate with agents tested in 96-well plates for 96 h. After addition of MTT solution, cells were incubated for additional 3 h at 37 °C, the insoluble formazan crystals obtained by centrifugation were dissolved in DMSO and the absorbance was read at 595 nm with the reference wavelength of 655 nm using BioRad 680XR plate reader (Hercules, CA, USA).

### Flow cytometry

Cultured cells were washed, aliquoted in three tubes and incubated with 8% Fc Receptor Blocking solution for 10 min. Cells in first tube were stained with anti-CD11b-FITC, anti-CD45-PerCP and anti-CD34-APC, in second tube with anti-CD64-FITC and in third tube with their respective isotypic controls for additional 20 min at room temperature in the dark. When used to test for cell viability of gated population, 7-AAD was added to tube containing anti-CD64 FITC for the last 2 min of incubation. Flow cytometry analyses were performed using FACS Calibur system (Becton Dickinson Immunocytometry Systems, San Jose, CA, USA). Cells were gated based upon forward and side scatter patterns and a total of 10,000 events were collected for each marker from this gated area. The obtained data were analyzed using CellQuest software and FlowJo v.10 platform. Mean fluorescence intensity (MFI) of the sample was calculated by subtracting MFI levels of isotypic controls from MFI levels of the cells stained with CD-specific antibodies. Percentage of positive cells was determined by measuring the fluorescence shift of distinct cluster of leukemic events.

For cell cycle analysis, cells were stained with propidium iodide (PI) solution (50 μg/mL of PI, 10 mM Tris, pH 8.0, 10 mM NaCl, 10 μg/mL RNase A, 0.1% Igepal) at + 4 °C for 20 min. DNA analysis was performed by collecting 10,000 events for each sample gated in order to eliminate aggregates and cell debris using FACSCalibur System, while representative histograms were created by using FlowJo (LLC, Ashland, OR, USA).

### Microscopic analysis

Samples were cytospun on microscopic slides using a StatSpin Cytofuge 2 (1000 rpm, 2 min) and left to dry overnight. Slides were stained with May-Grünwald stain (50% working solution, 5 min) and, subsequently, Giemsa stain (10% working solution, 20 min). Morphology was examined using AxioVert 200 microscope and images were photographed using Axiocam MRc 5 camera and ZEN software, blue edition (Carl Zeiss AG, Oberkochen Germany).

### Automated image analysis

Microscopic images were analyzed using CellProfiler and CellProfiler Analyst software as previously described [22]. Briefly, raw color images were split to red, green and blue channels, intensity was rescaled and segmentation was performed on green channel identifying nuclei as primary, whole cells as secondary and cytoplasm as tertiary objects. Cytological profile was generated for every cell using “Measure Object Size Shape” module in CellProfiler. Subsequent analysis was performed using CellProfiler Analyst in order to classify cells into three phenotypic categories: macrophage-like, negative and dead cells. A Random Forrest Classifier model used for this purpose was trained by manually classifying 812 randomly fetched objects (cells) and was evaluated by constructing confusion matrix. CellProfiler pipelines, CellProfiler classifier model and training set are available on request.

### RNA-sequencing and data analysis

AML sample Pt 14 and U937 cells were cultured as described above and treated with 0.4 and 0.2 mM AICAr, respectively, for 24 h. RNA isolation, sample quality assessment, RNA library preparation, sequencing and partial data analysis (raw data quality control, trimming, mapping, aligning and calculating gene hit counts) were conducted at GENEWIZ, Inc. (South Plainfield, NJ, USA). The RNA samples were quantified using Qubit 2.0 Fluorometer (Life Technologies, Carlsbad, CA, USA) and RNA integrity was checked with Agilent TapeStation (Agilent Technologies, Palo Alto, CA, USA). NEBNext Ultra RNA Library Prep Kit for Illumina (NEB, Ipswich, MA, USA) was used for preparation of cDNA libraries and sequencing was performed on the Illumina HiSeq 4000 or equivalent instrument in High Output Mode (2 × 150 Paired End configuration).

After investigating the quality of the raw data, sequence reads were trimmed using Trimmomatic v.0.36 and mapped to the reference genome *Homo sapiens* GRCh38 using the STAR aligner v.2.5.2b. Unique gene hit counts were calculated from BAM files by using feature Counts from the Subread package v.1.5.2.

Downstream differential expression analysis and functional analyses were performed entirely in R. To normalize counts and perform differential expression analysis DESeq2 was used (v. 1.24.0) with *p*-value threshold < 0.05 and log2 fold change threshold > 1. ClusterProfiler package [23] was used to further analyze differentially expressed genes by gene set enrichment analysis (GSEA) on KEGG gene sets. The GSEA algorithm was run using log fold change values as the gene-level statistics, 100,000 random permutations.

Publicly available datasets TCGA AML [24], GSE15434 [25] and OHSU BeatAML [26] were used for expression analysis of uridine monophosphate synthase (UMPS) and DHODH in AML patients. Data was accessed through Bloodspot [27] and cBioPortal [28]. Difference in gene expression levels between different groups were assessed using ANOVA with post hoc Tukey test and *p*-values < 0.05 were considered statistically significant. Correlation in gene expression was assessed using both Pearson’s and Spearman’s correlation coefficients and results in which both *p* < 0.05 and q < 0.05 were considered statistically significant.

### Statistical analysis

Apart from sequencing data, all other data are presented as mean ± standard error of the mean (S.E.M). Difference between groups was determined using ANOVA with post-hoc Dunnet test for comparison of groups to control group and *p*-values < 0.05 were considered statistically significant. Statistical analysis was performed using GraphPad Prism 6.0.

## Results

### AICAr decreases cell viability in primary AML samples

**Fig. 1:**
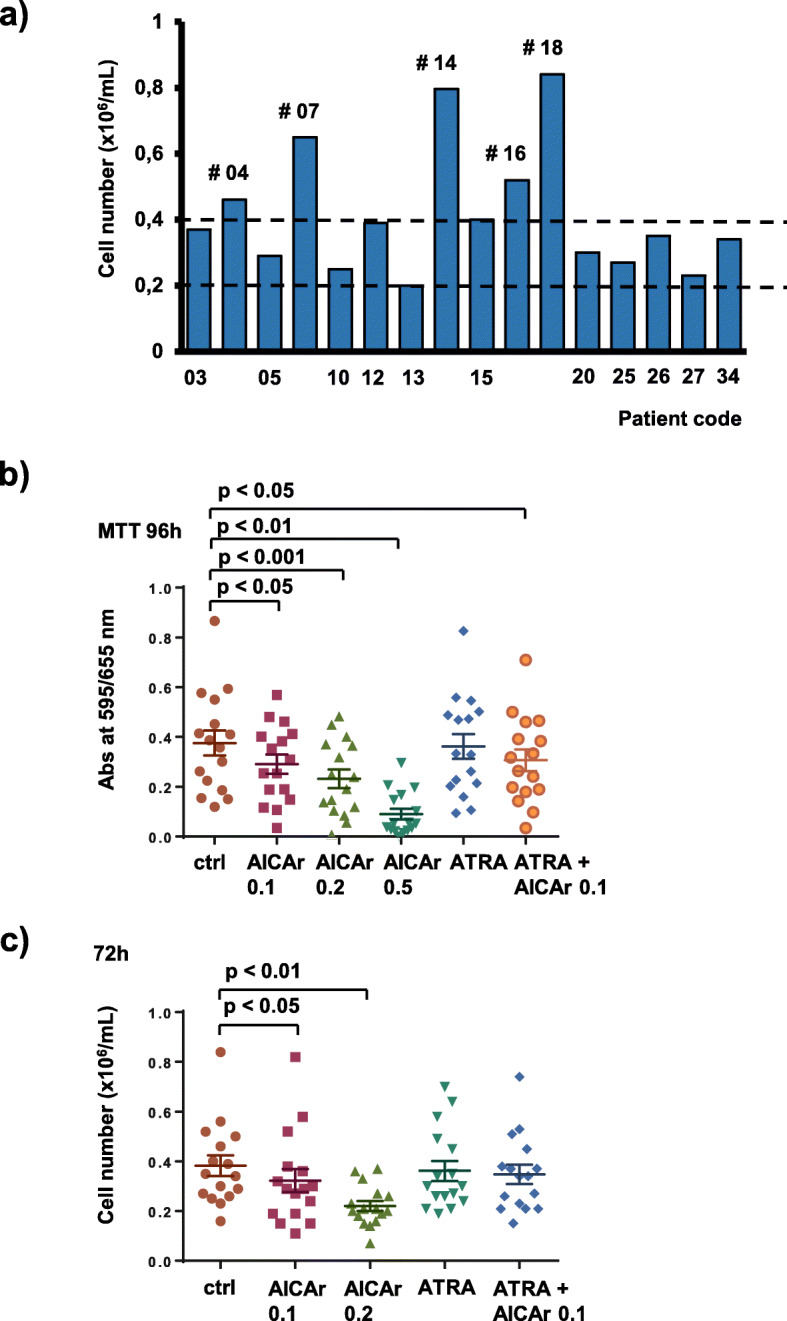
AICAr decreases cell viability in primary AML samples. Bone marrow samples of 16 AML patients were seeded at concentration 0.4 × 10^6^/mL in medium supplemented with 50 ng/mL IL-3, IL-6, SCF and FLT3L and incubated with agents tested. **a**) The number of viable control cells in patient samples after 72 h as determined by trypan blue exclusion. **b**) Cells were incubated in triplicates with AICAr (0.1–0.5 mM), ATRA (1 μM), and combination of AICAr (0.1 mM) and ATRA (1 μM) in 96-well plates and MTT assay was performed after 96 h. The mean value of MTT assay is presented for every patient. Results are mean ± S.E.M, *N* = 16 **c**) Primary samples were incubated with agents tested in 12-well plates and the number of viable cells was determined after 72 h by trypan blue exclusion. Results are mean ± S.E.M, *N* = 16. Statistical significance was determined using one-way ANOVA and post-hoc Dunnett test

To screen first for the possible antiproliferative effects of AICAr in primary AML samples, an initial set of bone marrow samples from 35 patients suffering from non-APL AML was collected. Mononuclear cells were isolated from bone marrow samples by density gradient centrifugation, allowed to adhere overnight on plastic, and the non-adherent cells were grown in vitro in RPMI medium with the addition of IL-3, IL-6, SCF and FLT3L. Without any drugs added, 16 samples had more than 0.2 × 10^6^ of viable cells per mL after 72 h of incubation (Fig. 1a). These samples were included in further analyses, and patient characteristics are presented in Supplementary Table 2.

The results of MTT assay showed that AICAr significantly reduced the viability of patient samples in a concentration-dependent manner (Fig. 1b). Knowing that AICAr exerts metabolic effects that may affect the results of MTT assay [29], the reduction in cell viability was confirmed by simple counting of trypan blue-negative cells on the hemocytometer (Fig. 1c). These results demonstrate that AICAr decreases cell viability in primary AML samples.

### AICAr and brequinar induce differentiation in a subset of primary AML samples that are resistant to ATRA

AICAr, brequinar and ATRA were then tested for the capability to induce differentiation in sixteen viable samples. As shown in Fig. 1a, only five of these samples exhibited proliferation in the absence of drugs, as judged by having the number of viable cells above plating density (more than 0.4 × 10^6^) after 72 h. Two out of these proliferating samples exhibited upregulation of the differentiation marker CD11b upon AICAr treatment; FAB-M2 and FAB-M4 (Pt 14 and Pt 07).

**Fig. 2:**
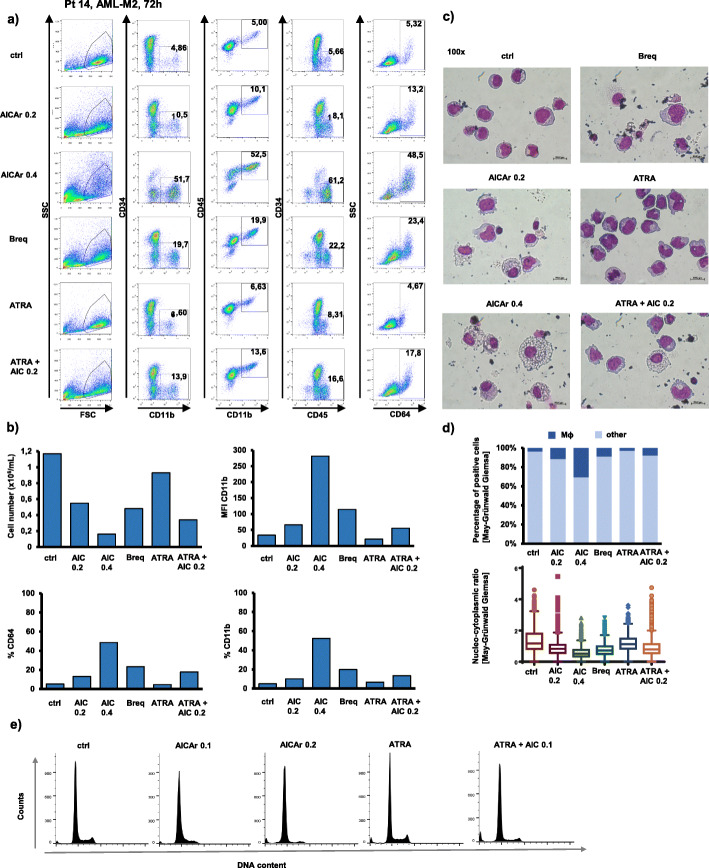
AICAr induces differentiation and accumulation of macrophage-like cells in a primary sample from AML-M2 patient (normal karyotype, *FLT3*wt *NPM1*wt, primary refractory). Bone marrow sample (Pt 14) was seeded at concentration 0.4 × 10^6^/mL in medium supplemented with 50 ng/mL IL-3, IL-6, SCF and FLT3L and incubated with AICAr (0.2 and 0.4 mM), ATRA (1 μM), combination of AICAr (0.2 mM) and ATRA (1 μM), or brequinar (500 nM) for 72 h. **a**) Flow cytometric analysis of CD11b^+^CD34, CD11b^+^CD45^+^, CD45^high^CD34^−^ and CD64^+^ populations. Percentage of cells in population of interest is indicated in respective gates. **b**) The number of viable cells was determined by trypan blue exclusion. Mean fluorescence intensity (MFI) of CD11b and percentage of CD64 and CD11b positive cells were determined by flow cytometry. **c**) May-Grünwald-Giemsa stained cytospin preparations (100x magnification). **d**) Proportion of macrophage-like cells in samples and nucleo-cytoplasmic ratio of cells determined using automated image analysis of at least 1000 cells per representative cytospin preparation (10x magnification). **e**) Cell cycle analysis of propidium-labeled cells. Results are representatives of three independent experiments

In one patient sample (Pt 14: FAB-M2, normal karyotype, *FLT3*wt *NPM1*wt, primary refractory), the addition of AICAr in concentrations that reduced the number of leukemia cells resulted in a pronounced increase in the expression of markers CD11b and CD64, as well as an increase in percentage of CD45^high^ cells, suggesting the differentiation of blasts to more mature forms (Fig. 2a & b). Immunophenotypic changes in AICAr-treated cells were accompanied by accumulation of cells that morphologically resembled macrophages as they show an increase in the cell size, the vacuolization of the cytoplasm and a decrease in nucleo-cytoplasmic ratio. The similar effects were observed in cells treated with brequinar, and no significant differentiative effects were seen in cells treated with ATRA (Fig. 2c & d). In addition, cell cycle analysis of propidium-labeled cells revealed a decrease in G_2_/M phase of AICAr-treated cells and inhibition of progression through the S-phase (Fig. 2e).

**Fig. 3:**
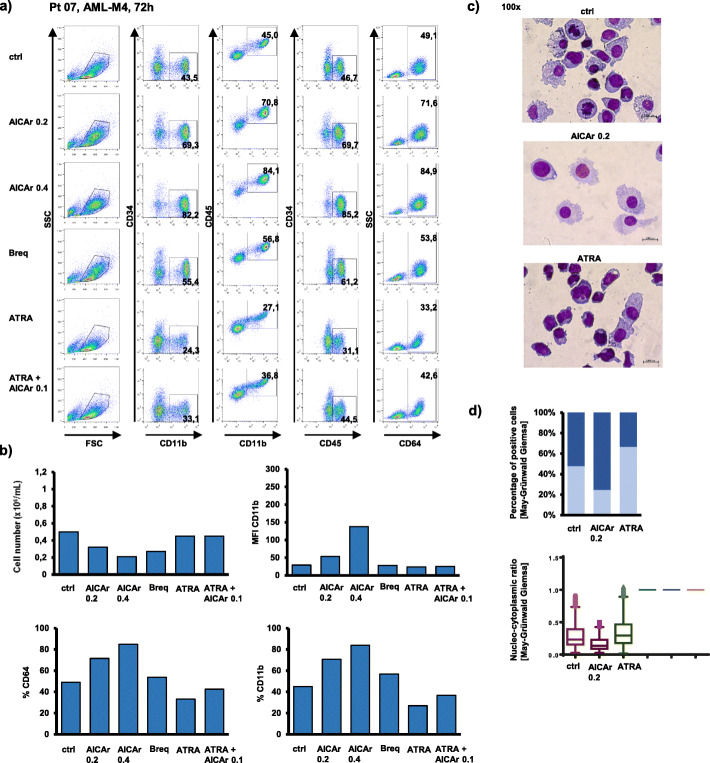
AICAr increases proportion of more mature monocytic cells in a primary sample from AML-M4 patient (normal karyotype, *FLT3*-ITD, *NPM1*mut). Bone marrow sample (Pt 07) was seeded at concentration 0.4 × 10^6^/mL in medium supplemented with 50 ng/mL IL-3, IL-6, SCF and FLT3L and incubated with AICAr (0.2 and 0.4 mM), ATRA (1 μM), combination of AICAr (0.1 mM) and ATRA (1 μM), or brequinar (500 nM) for 72 h. **a**) Flow cytometric analysis of CD11b^+^CD34^−^, CD11b^+^CD45^+^, CD45^high^CD34^−^ and CD64^+^ populations. Percentage of cells in population of interest is indicated in respective gates. **b**) The number of viable cells was determined by trypan blue exclusion and mean fluorescence intensity (MFI) of CD11b was determined by flow cytometry. Results are a representative experiment of three independent experiments. **c**) May-Grünwald-Giemsa stained cytospin preparations (100x magnification) from representative of two independent experiments. **d**) Proportion of macrophage-like cells and nucleo-cytoplasmic ratio of cells in representative experiment determined using automated image analysis of at least 500 cells per cytospin preparation (10x magnification)

In a sample of more mature myelomonocytic cells (Pt 07: FAB-M4, normal karyotype, *FLT3*-ITD, *NPM1*mut), the basal level of CD11b positive cells was higher, but further increased in the presence of both AICAr and brequinar (Fig. 3a). Moreover, the increase in the expression of CD11b was associated with an increase in CD64 expression, a decrease in cell number (Fig. 3b) and morphological changes associated with differentiation into macrophage-like cells (Fig. 3c). Again, no increase in the level of CD11b was seen in cells treated with ATRA.

We concluded that AICAr-induced differentiation in a subset of primary non-APL AML blasts had no correlation with either FAB classification, cytogenetics or mutational status of *FLT3* or *NPM1*. AICAr-sensitive primary AML samples differentiated in the presence of brequinar, but were resistant to differentiative effects of ATRA. To further test if there is any connection between the expression of UMPS and DHODH, which are presumed molecular targets of AICAr and brequinar, and patients cytogenetic and genetic signature, we performed the analysis on publicly available datasets. Expression analysis of DHODH and UMPS in TCGA and GSE15434 datasets showed no significant differences in the expression of these genes in AML patients depending on the cytogenetics or mutational status in *CEBPA*, *FLT3* or *NPM1* (Supplementary Fig. 1A), and the expression of both *DHODH* and *UMPS* genes was inversely correlated with the expression of *ITGAM* (CD11b) in TCGA and OHSU BeatAML datasets (Supplementary Fig. 1B).

### AICAr treatment changes the expression of genes involved in regulation of cell cycle and pyrimidine metabolism in responsive AML patient

**Fig. 4:**
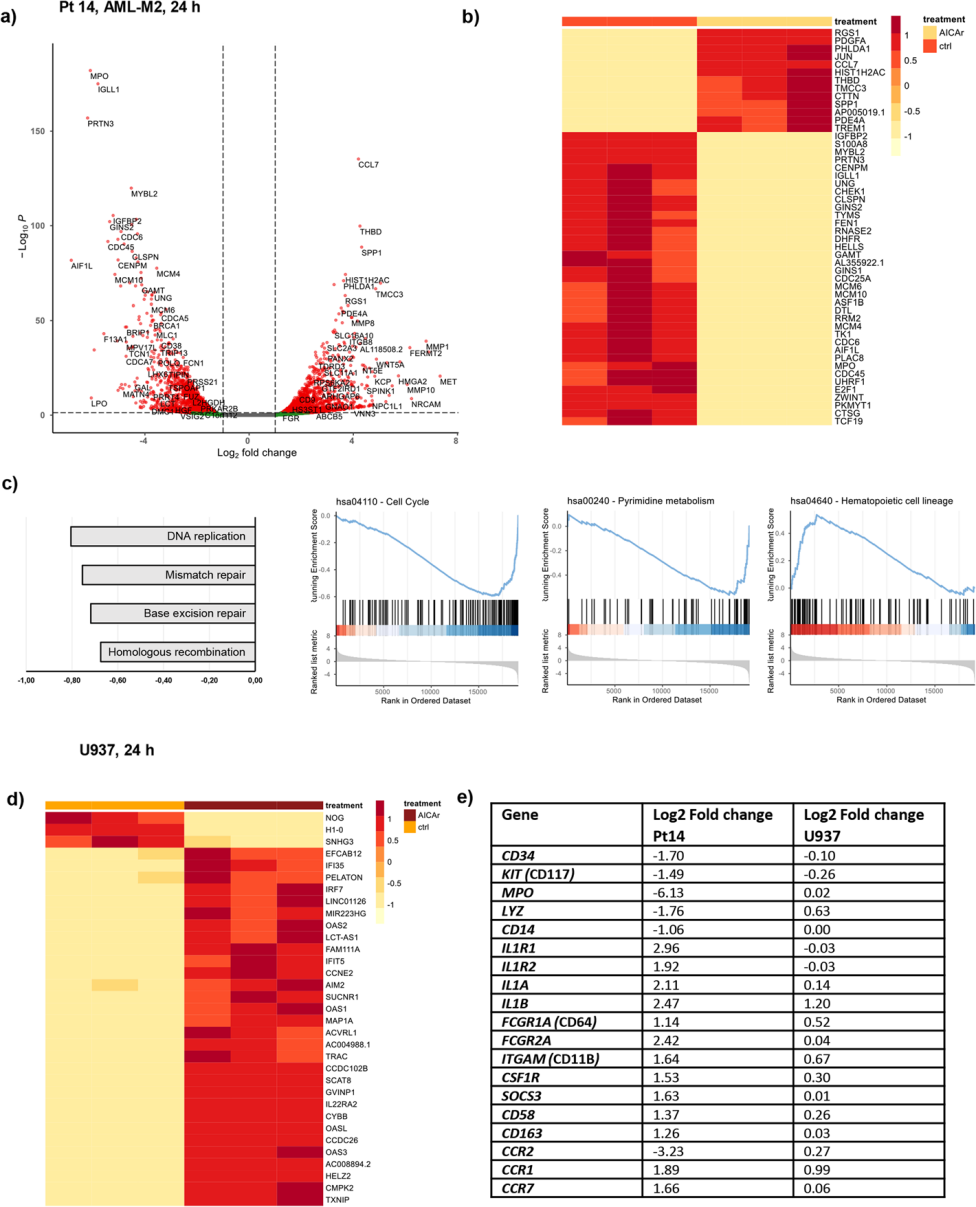
Differential gene expression in primary sample from AML-M2 patient (normal karyotype, *FLT3*wt *NPM1*wt, primary refractory) and U937 cells in response to AICAr. Bone marrow sample from the AML patient (Pt 14) in which AICAr induced differentiation in vitro and U937 cells were seeded in triplicates and treated with AICAr for 24 h. Differential gene expression was determined using RNA sequencing and DESeq2 analysis was performed. **a**) Volcano plot. The red dots: |log2 FC| ≥ 1, *p*.adjusted < 0.05; the green dots: |log2 FC| ≥ 1, *p*.adjusted > 0.05; the black dots: |log2 FC| ≤ 1, *p*.adjusted > 0.05. **b**) Heatmap from hierarchical clustering of top 50 significant genes ranked by p.adjusted value between untreated and AICAr-treated primary samples. **c**) Gene Set Enrichment Analysis (GSEA) for KEGG pathways: Top 4 most enriched pathways and GSEA plots (score curves) for pathways Cell Cycle, Pyrimidine Metabolism and Hematopoietic Cell Lineage. *p*.adjusted < 0.01. **d**) Heatmap from hierarchical clustering of top 30 significant genes ranked by *p*.adjusted value between control and AICAr-treated U937 cells. **e**) Log2Fold changes in the expression of genes associated with immature cells, monocytes and macrophages in primary sample and U937 cells

To elucidate the effects of AICAr on gene expression in primary AML cells, we performed RNA sequencing of control and AICAr-treated cells isolated from bone marrow of AML patient (Pt 14) in which AICAr induced the strongest differentiative response. RNA was isolated from cells that were cultured in triplicates and treated with or without 0.4 mM AICAr for 24 h. As shown in Fig. 4a, genes associated with monocyte/macrophage differentiation (*SPP1*, *THBD*, *CCL7*...), and those associated with neutrophil function (*MPO*, *PRTN3*…) were among top significant genes regulated by AICAr. Furthermore, hierarchical clustering of top 50 significant genes revealed a large proportion of genes associated with DNA replication and cell cycle (*PHLDA1*, *MCM*, *CLSPN*, *CHEK1*, *MYBL2*, *ZWINT*, *CDC6*, *CDC25A*) as well as several genes related to purine and pyrimidine metabolism (*TK1*, *TYMS*, *DHFR*) (Fig. 4b). In addition, gene set enrichment analysis (GSEA) on KEGG database was performed and complete results are presented in Supplementary Table 3. As shown in Fig. 4c, GSEA on KEGG pathways confirmed that AICAr treatment induced a strong downregulation of cell cycle and pyrimidine metabolism pathways together with a strong upregulation of gene set involved in hematopoietic cell lineage.

To compare the transcriptional profile of AICAr-treated primary AML cells with the effects of AICAr in monocytic U937 cell line in which AICAr-mediated differentiation was first observed [12], RNA sequencing was performed in control and AICAr-treated U937 cells (Fig. 4d). As shown in Fig. 4e, an increase in the expression of genes associated with macrophage differentiation (e.g. *CSF1R, SOCS3, CD163, CCR1, CCR7*…) followed by a decrease in the expression of markers of immature cells (*CD34* and *KIT/CD117*) or monocytes (*CCR2*) was observed in both primary AML cells and U937 cell line. In addition, list of common gene expression changes (Supplemental Table 4) included genes associated with cytokines, chemokines and their receptors (*IL1B, IL1RN, CCL2, CCL3, CCR1*), metabolism (*MAN1B1-AS1, IPP, PTGS2, ABHD4...*) and cell cycle progression, DNA repair and apoptosis (*CDKN1A, PPP1R10, PANO1…)*.

We conclude that RNA-seq data obtained in primary AML blasts treated with AICAr are in agreement with our previous data showing that AICAr-induced differentiation of AML cell lines depended on cell cycle arrest through pyrimidine depletion [13].

## Discussion

AICA-ribonucleoside (AICAr) is an exogenous substance which, after entering cells, becomes phosphorylated into AICA-ribonucleotide (AICAR or ZMP), and AICAR is an evolutionary conserved cellular intermediate in de novo purine synthesis that accumulates in Lesch-Nyhan syndrome [30]. Although both AICAr and metformin have been widely used as AMPK agonists in studies related mostly to insulin signaling and metabolism, there are many recent studies showing that some of their effects on cell cycle, growth and metabolism are actually AMPK-independent [31, 32]. Because of structural similarities with adenosine, AICAr was first used to prevent cardiovascular complications in patients undergoing coronary artery bypass surgery [33], but then became popular as an “exercise in a pill” as the study in mice revealed that AICAr increased endurance and mimicked the effects of aerobic exercise [34]. The most recent interest in AICAr was gained by studies reporting anti-tumor effects of metformin and other AMPK agonists in cancer [35]. Cytotoxic effects of AICAr in vitro have been demonstrated in several hematological malignancies at concentrations that are well tolerated when achieved in plasma after intravenous injection [36–38], and results of clinical trial testing the effects in B cell chronic lymphocytic leukemia showed that AICAr had an acceptable safety profile and antileukemic activity in patients with poor prognosis. The pharmacokinetic analysis performed during that clinical trial demonstrated that at the maximum tolerated dose of 210 mg/kg, the plasma concentration of AICAr and its metabolite ZMP corresponded to 0.9 mM [39]. The results of the present study show that lower concentrations of AICAr exert significant antiproliferative effects in primary AML samples.

The antiproliferative effects of AICAr in AML cell lines were previously proved to be AMPK-independent [12]. Both AICAr and metformin induced apoptosis, but only AICAr induced differentiation that depended on the inhibition of UMP-synthase and was completely overcame by addition of uridine [13]. AICAr shares the same mechanisms of action with well-known inhibitors of de novo pyrimidine synthesis that have been previously shown to induce uridine-dependent leukemia differentiation, such as DHODH inhibitors brequinar [10] and leflunomide [40], or UMP synthase inhibitor pyrazofurin [10]. In AML cell lines, low concentrations of AICAr and brequinar were shown to exert synergistic effects on both cell cycle arrest in S-phase and cellular differentiation, and the effects of both agents depended on the activation of DNA damage ATR/Chk1 signaling pathway [13]. In this study, we present evidence that both drugs can induce an increase in the expression of differentiation markers and morphological changes associated with maturation in samples isolated from patients with AML, and these effects do not correlate with either FAB classification or mutational status of *NPM1* and *FLT3*-ITD. However, the effects of both AICAr and brequinar on differentiation of primary AML blasts required proliferation in conditions in vitro, and this would be expected from the proposed mechanism of AICAr- and brequinar-mediated differentiation that depends on the lack of pyrimidine synthesis and the cell cycle arrest.

There is a considerable interest toward the development of new inhibitors of DHODH as a potential option for differentiation therapy of AML. Brequinar was tested in clinical trials for solid tumors, but myeloid suppression and severe adverse reactions limited its clinical application. In spite of known toxicity, a currently ongoing phase 1b/2a clinical trial (NCT03760666) is trying to assess its safety and efficacy in patients with relapsed/refractory AML [20]. Leflunomide and teriflunomide are FDA-approved for rheumatoid arthritis and multiple sclerosis, respectively, but they have failed to receive FDA approval for cancer [41]. The novel DHODH inhibitor BAY 240234 is currently under phase 1 clinical trial (NCT03404726) for myeloid malignancies, and preclinical data showed differentiative effects in several AML cell lines, including THP-1, and three subcutaneous AML xenografts in vivo [18]. Another novel DHODH inhibitor, ASLAN003 is currently being evaluated in a phase 2a trial (NCT03451084) in AML and preclinical data on primary AML blasts grown under similar conditions as the ones used in our study revealed sensitivity in six out of fourteen AML samples [19]. In study by Zhou at al., sensitivity was defined as an increase ≥15% in any of markers CD11b, CD14, CD13 or CD33 [19], while our present study defines response as a significant increase in the level of CD11b. However, both studies agree that differentiation of AML in response to ASLAN003, AICAr or brequinar is independent from FAB subtype or mutational status of *FLT3* or *NPM1*.

Results of our study show that sensitivity to AICAr correlates well with sensitivity to brequinar in primary AML samples. Although there is a potential mechanistic link in the activity of pyrimidine pathway and differentiation blockade in AML patients, our analysis of the expression of DHODH and UMPS further suggests that patients who could potentially benefit from the modulation of the activity of pyrimidine synthesis pathway are not easily identifiable using conventional diagnostic markers in AML. Our previous work provided the link between the defect in pyrimidine synthesis, cell cycle arrest, ATR/Chk1 pathway and cellular differentiation [13] and present data obtained by RNA sequencing, albeit limited to just one patient sample, point to the similar direction. In contrast, none of the samples that responded to inhibitors of pyrimidine synthesis differentiated in the presence of ATRA. These results are in accordance with our previous data demonstrating that the effects of AICAr on metabolism and cell cycle progression differ from the effects of ATRA and that these two drugs act via independent mechanisms [13]. Further investigations of the pleiotropic effects of pyrimidine inhibition will be required to fully understand the mechanism and to identify the subtype of non-APL AML patients that may benefit from this differentiation therapy, but it is possible that the mechanism of differentiation more closely resembles that of low doses of cytotoxic drugs, and involves pathways that regulates cell cycle, DNA damage response and apoptosis.

## Conclusions

The results of our present study demonstrate that AICAr induces differentiation in a subset of non-APL AML blasts isolated from bone marrow of patients suffering from AML, and these effects do not correlate with FAB classification or mutational status of *FLT3* or *NPM1*, but correlate with sensitivity to well-known, potent DHODH inhibitor.

## Supplementary information

**Additional file 1 Supplementary Table 1.** Reagents and resources used.

**Additional file 2 Supplementary Table 2**. Patient characteristics.

**Additional file 3 Supplementary Table 3.** Results of GSEA analysis on KEGG gene sets.

**Additional file 4 Supplementary Table 4.** Common gene expression changes of control and AICAr-treated bone marrow sample (Pt14) and U937 cells in relation to their respective untreated controls (selected from the results of the differential gene expression analysis conducted by GENEWIZ).

**Additional file 5 Supplementary Figure 1.** Expression of DHODH and UMPS in AML patient samples. A) Publicly available datasets TCGA and GSE15434 (accessed through Bloodspot database) with a total of 413 patients were used to compare expression levels of DHODH and UMPS in primary AML samples in accordance to their cytogenetics and mutational status of *CEBPA*, *FLT3* and *NPM1.* B) Gene co-expression analysis between *DHODH* or *UMPS* and *ITGAM* (CD11b) was performed on publicly available datasets TCGA and OHSU (accessed through cBioPortal database) which jointly comprise 567 samples.

## Data Availability

The datasets generated and analysed during the current study are available in the Array Express Archive repository, https://www.ebi.ac.uk/arrayexpress/experiments/E-MTAB-9209/, https://www.ebi.ac.uk/arrayexpress/experiments/E-MTAB-9537/

## Abbreviations

AICAr: 5-aminoimidazole-4-carboxamide ribonucleoside
AICAR: 5-aminoimidazole-4-carboxamide ribotide (ZMP)
AML: Acute myeloid leukemia
AML1-ETO: Acute myeloid leukemia 1- Eight-twenty-one oncoprotein
AMP: Adenosine monophosphate
AMPK: AMP-activated kinase
APL: Acute promyelocytic leukemia
ATO: Arsenic trioxide
ATR/Chk1: Ataxia telangiectasia and Rad3-related protein/Checkpoint kinase 1
ATRA: All-*tran*s retinoic acid
BCR-ABL: Breakpoint cluster region-Abelson murine leukemia viral oncogene homolog 1
CBFB/MYH11: Core-binding factor subunit beta/Myosin heavy chain 11
CCL7: C-C motif chemokine ligand 7
CDC25A: Cell division cycle 25A
CDC6: Cell division cycle 6
CHEK1: Checkpoint kinase 1
CLSPN: Claspin
DHFR: Dihydrofolate reductase
DHODH: Dihydroorotate dehydrogenase
DMSO: Dimethyl sulfoxide
FLT3: FMS-related tyrosine kinase 3
GSEA: Gene set enrichment analysis
IDH: Isocitrate dehydrogenase
ITD: Internal tandem duplication
KEGG: Kyoto Encyclopedia of Genes and Genomes
MCM: Minichromosome maintenance complex
MLL-AF4/AF9: Lysine methyltransferase 2A- AF4/FMR2 family member 1/MLLT3 super elongation complex subunit
MPO: Myeloperoxidase
MTHFD2: Methylenetetrahydrofolate dehydrogenase-cyclohydrolase 2
MTT: 3-(4,5-dimethylthiazol-2-yl)-2,5-diphenyltetrazolium bromide
MYBL2: MYB proto-oncogene like 2
NPM1: Nucleophosmin 1
PHLDA1: Pleckstrin homology like domain family A member 1
PML/RARa: Promyelocytic leukemia/Retinoic acid receptor α
PRTN3: Proteinase 3
SCF: Stem cell factor
SPP1: Secreted phosphoprotein 1
THBD: Thrombomodulin
TK1: Thymidine kinase 1
TYMS: Thymidylate synthetase
UMP: Uridine monophosphate
ZMP: 5-aminoimidazole-4-carboxamide ribotide (AICAR)
ZWINT: ZW10 interacting kinetochore protein
2-HG: R-2-hydroxyglutarate
7-AAD: 7-aminoactinomycin D
