# Supplementary material for: 5-aminoimidazole-4-carboxamide ribonucleoside induces differentiation in a subset of primary acute myeloid leukemia blasts
Source: BMC Cancer. 2020 Nov 11;20:1090. doi: 10.1186/s12885-020-07533-6 (PMC7657321; doi:10.1186/s12885-020-07533-6)
Supplement: Supplementary file 1 — Additional file 1 Supplementary Table 1. Reagents and resources used. [file 12885_2020_7533_MOESM1_ESM.docx]

**Supplementary Table 1. Reagents and resources used**

| **Reagent or RESOURCE** | **Source** | **Identifier** |
| --- | --- | --- |
| **Antibodies** | | |
| IgG1-FITC (clone 679.1Mc7) | Immunotech BeckmanCoulter, Marseille France | A07795 |
| IgG1-APC (MOPC-21) | BD Biosciences, San Jose, CA, USA | 555751 |
| IgG1-PerCP (559425) | BD Biosciences, San Jose, CA, USA | 559425 |
| CD11b-FITC (clone Bear1) | Immunotech BeckmanCoulter, Marseille France | IM0530 |
| CD64-FITC (clone 22) | Immunotech BeckmanCoulter, Marseille France | B49186 |
| CD34-APC (8G12) | BD Biosciences, San Jose, CA, USA | 345804 |
| CD45-PercP (2D1) | BD Biosciences, San Jose, CA, USA | 345809 |
| **Chemicals and peptides** | | |
| AICAr | Sigma, St. Louis, MO, USA | A9978 |
| ATRA | Calbiochem, San Diego, CA, USA | 554720 |
| brequinar | Sigma, St. Louis, MO, USA | SML0113 |
| propidium iodide | Sigma, St. Louis, MO, USA | P4170 |
| thyazolil blue tetrazolium bromide (MTT) | Sigma, St. Louis, MO, USA | M2128 |
| 7-AAD staining solution | Miltenyi Biotec GmbH, Bergisch Gladbach, Germany | 130-111-568 |
| Human TruStain FcX™ Fc Receptor Blocking solution | BioLegend, San Diego, CA, USA | 422302 |
| NycoPrep 1.077 solution | Axis-Shield PoC AS , Oslo, Norway | 1114741 |
| rhIL-3 | R&D Systems, Minneapolis, MN, USA | 203-IL |
| rhIL-6 | R&D Systems, Minneapolis, MN, USA | 206-IL |
| rhFlt3/Flk2 ligand | R&D Systems, Minneapolis, MN, USA | 308-FK |
| rhSCF | R&D Systems, Minneapolis, MN, USA | 255-SC |
| **Cell culture** | | |
| RPMI Medium 1640 | Gibco/Invitrogen, Grand Island, NY, USA | 42401-018 |
| fetal bovine serum (FBS) | Gibco/Invitrogen, Grand Island, NY, USA | 10270-106 |
| L-Glutamine 200mM | Gibco/Invitrogen, Grand Island, NY, USA | 25030-024 |
| penicillin/streptomycin | Gibco/Invitrogen, Grand Island, NY, USA | 15070-063 |
| **Cell lines** | | |
| U937 | ECACC, Porton, Salisbury, UK | 85011440 |
| **Software** | | |
| CellQuest | Becton Dickinson Immunocytometry Systems, San Jose, CA, USA | N/A |
| ModFit LT | Verity Software House | www.vsh.com/products/mflt/index.asp |
| FlowJo v10 | FlowJo LLC, Ashland, OR, USA | flowjo.com |
| GraphPad Prism 6 | GraphPad Software | graphpad.com |
| R Studio | R Studio, Boston, MA, USA | rstudio.com |
| ZEN lite Blue edition | Carl Zeiss AG, Oberkochen Germany | www.zeiss.com/microscopy/int/products/microscope-software/zen-lite.html |
| Cell Profiler | Broad Institute, Cambridge, MA, USA | cellprofiler.org |
| Cell Profiler-Analyst | Broad Institute, Cambridge, MA, USA | cellprofiler.org/cp-analyst/ |
