# Supplementary material for: 5-aminoimidazole-4-carboxamide ribonucleoside induces differentiation in a subset of primary acute myeloid leukemia blasts
Source: BMC Cancer. 2020 Nov 11;20:1090. doi: 10.1186/s12885-020-07533-6 (PMC7657321; doi:10.1186/s12885-020-07533-6)
Supplement: Supplementary file 2 — Additional file 2 Supplementary Table 2. Patient characteristics. [file 12885_2020_7533_MOESM2_ESM.docx]

**Supplementary Table 2. Patient characteristics**

| **Patient code** | **age** | **FAB (cytology)** | **WBC** | **cytogenetics** | **molecular markers** | **blasts (%, cytology)** | **immunophenotype** |
| --- | --- | --- | --- | --- | --- | --- | --- |
| 03 | 21-30 | M4 | 13.3 | 45, X -X, t(3;14), t(8;21)/46, XX; RUNX1/RUNX1T1, | FLT3-ITD, AML1-ETO | 50 | MPO-CD4-CD13+CD14-CD15- CD33+CD34+CD61-CD64-lysozyme-CD117+HLADR+TdT-CD3-CD7-CD19-CD56+CD79a-CD235- |
| 04 | 61-70 | M2 | 53.4 | 46, XX; *FISH negative: MLL* | *negative: AML1-ETO, FLT3* | 88 | MPO+CD4-CD13+CD14-CD15-CD33+CD34+CD61-CD64-lysozyme-CD117+HLADR-TdT-CD3-CD7-CD19-CD56-CD79a-CD235- |
| 05 | 51-60 | M4 | 11.3 | Complex karyogram, inv(16) | CBFB/MYH11 | 34 | MPO-CD4+CD13+CD14+CD15+ CD33+CD34+CD61-CD64+lysozyme+ CD117+ HLADR+TdT-CD3-CD7-CD19-CD56-CD79a-CD235- |
| 07 | 51-60 | M4 | 30.5 | 46, XX | FLT3-ITD, NPM1 | 34 | MPO-CD4+CD13+CD14+CD15+ CD33+CD34-CD61-CD64+lysozyme+ CD117+HLADR+TdT-CD3-CD7-CD19-CD56-CD79a-CD235- |
| 10 | 21-30 | M2 | 2.6 | 46, XX | *negative: FLT3, NPM1* | 35 | MPO+CD4-CD13+CD14-CD15-CD33+CD34+CD61-CD64-lysozyme-CD117+HLADR+TdT-CD3-CD7+CD19-CD56-CD79a-CD235- |
| 12 | 21-30 | M2 | 12.3 | 45, X - X, +8, t(8;21)/46, XX; RUNX1/RUNX1T1, | AML1-ETO | 73 | MPO+CD4+CD13+CD14-CD15+ CD33+CD34+CD61-CD64-lysozyme-CD117+HLADR+TdT-CD3-CD7-CD19+ CD56-CD79a-CD235- |
| 13 | 61-70 | M2 | 8.2 | 45, X, -Y, t(8;21)(q22;q22) /46,XY; RUNX1/RUNX1T1 | AML1-ETO | 28 | MPO-CD4-CD13+CD14-CD15-CD33+CD34+CD61nt CD64-lysozyme-CD117+HLADR+TdT-CD3-CD7-CD19+CD56-CD79a-CD235- |
| 14 | 31-40 | M2 | 25.8 | 46, XY | *negative: FLT3, NPM1, BCR-ABL* | 20 | MPO-CD4-CD13+CD14-CD15-CD33+CD34+CD61-CD64-lysozyme-CD117+HLADR+TdT-CD3-CD7-CD19-CD56-CD79a-CD235- |
| 15 | 31-40 | M4 | 26.8 | Complex karyogram, t(8;21); RUNX1/RUNX1T1 | FLT3-ITD, AML1-ETO | 30 | MPO+CD4-CD13+CD14-CD15-CD33+CD34+CD61-CD64-lysozyme-CD117+HLADR+TdT-CD3-CD7-CD19+CD56-CD79a-CD235- |
| 16 | 31-40 | M2/AML-MRC | 1.7 | 46, XY | NPM1 | 28 | MPO+CD4-CD13+CD14-CD15-CD33+CD34-CD61-CD64-lysozyme-CD117+HLADR+TdT-CD3-CD7+CD19-CD56-CD79a-CD235- |
| 18 | 61-70 | M5a, therapy related | 106.9 | 45, XY, t(4;11)(q21;q23), -21 | MLL-AF4 | 71 | MPOnt CD4+CD13+CD14-CD15+ CD33+CD34-CD61-CD64+lysozyme+ CD117+HLADR+TdT-CD3-CD7+CD19+ CD56-CD79a-CD235- |
| 20 | 51-60 | M4 | 84 | MLL rearrangement | *negative: MLL-AF9, CBFB/MYH11* | 87 | MPO+CD4+CD13+CD14+CD15+ CD33+CD34-CD61-CD64+lysozyme+ CD117-HLADR+TdT-CD3-CD7-CD19-CD56-CD79a-CD235- |
| 25 | 51-60 | M4 | 84.8 | *FISH negative:* *inv(16), MLL* | FLT3-ITD, *negative: AML1-ETO* | 93 | MPO+CD4-CD11b-CD13+CD14-CD15-CD33+CD34+CD61-CD64- lysozyme nt CD117+HLADR+TdT-CD3-CD7+CD19+ CD56-CD79a-CD235- |
| 26 | 21-30 | M4Eo | 62.7 | 46, XY, inv(16) | CBFB/MYH11 | 66 | MPO-CD4+CD11b-CD13+CD14-CD15-CD33+CD34+CD61-CD64-lysozyme-CD117+HLADR+TdT-CD3-CD7-CD19-CD56- CD79a-CD235- |
| 27 | 21-30 | M2 | 133.9 | 46, XX, inv(16)(p13;q22)/46, XX | CBFB/MYH11 | 70 | MPO+CD4+CD11b+CD13+CD14- CD15+ CD33+CD34+CD61-CD64-lysozyme-CD117+HLADR+TdT-CD3-CD7-CD19-CD56- CD79a-CD235- |
| 34 | 71-80 | M2 | 31.2 | 46, XX; *FISH negative:* *t(15;17), t(8;21), t(9;22), del 6q21, MLL* | FLT3-ITD  *negative: PML-RARA* | 82 | MPO+CD4+CD11b-CD13+CD14-CD15-CD33+CD34-CD61-CD64-lysozyme-CD117+ HLADR-TdT-CD3-CD7-CD19-CD56-CD79a-CD235- |

nt – not tested
