# Supplementary material for: 5-aminoimidazole-4-carboxamide ribonucleoside induces differentiation in a subset of primary acute myeloid leukemia blasts
Source: BMC Cancer. 2020 Nov 11;20:1090. doi: 10.1186/s12885-020-07533-6 (PMC7657321; doi:10.1186/s12885-020-07533-6)
Supplement: Supplementary file 3 — Additional file 3 Supplementary Table 3. Results of GSEA analysis on KEGG gene sets. [file 12885_2020_7533_MOESM3_ESM.docx]

**Supplementary Table 3. Results of GSEA analysis on KEGG gene sets**

| **ID** | **Description** | **setSize** | **Enrichment**  **Score** | **NES** | **pvalue** | **p.adjust** | **qvalues** | **rank** | **leading_edge** | **core_enrichment** |
| --- | --- | --- | --- | --- | --- | --- | --- | --- | --- | --- |
| **hsa03030** | DNA replication | 36 | -0,8048 | -2,6711 | 0,0019 | 0,0068 | 0,0032 | 3263 | tags=89%, list=17%, signal=74% | 5981/84153/5424/6117/10714/79621/6742/54107/5558/5982/5984/5426/4174/5985/5111/6119/23649/5983/5425/1763/10535/3978/4172/5422/4171/4176/2237/4173/5557/5427/4175 |
| **hsa03430** | Mismatch repair | 23 | -0,7537 | -2,2648 | 0,0020 | 0,0068 | 0,0032 | 3866 | tags=83%, list=20%, signal=66% | 6118/5981/5424/6117/10714/4292/6742/2956/5982/5984/5985/5111/6119/4436/5983/5425/3978/9156 |
| **hsa03410** | Base excision repair | 32 | -0,7182 | -2,3310 | 0,0019 | 0,0068 | 0,0032 | 2027 | tags=47%, list=11%, signal=42% | 252969/328/54107/3146/142/5426/5111/4913/5425/3978/55247/2237/7374/5427 |
| **hsa03440** | Homologous recombination | 41 | -0,6742 | -2,3085 | 0,0019 | 0,0068 | 0,0032 | 4932 | tags=73%, list=26%, signal=54% | 11073/29935/84142/7156/79728/4361/6118/79184/7517/5424/675/5932/6117/10714/5892/6742/5890/580/25788/641/5889/6119/5425/146956/672/5888/7516/8438/83990 |
| **hsa03460** | Fanconi anemia pathway | 54 | -0,6508 | -2,3280 | 0,0019 | 0,0068 | 0,0032 | 4014 | tags=63%, list=21%, signal=50% | 545/548593/5395/2188/55120/57697/6118/675/9894/6117/91442/2178/4292/80233/7398/100526739/201254/2189/2177/641/5889/2175/6119/80010/2187/29089/55215/116028/146956/378708/672/5888/83990 |
| **hsa03008** | Ribosome biogenesis in eukaryotes | 77 | -0,6352 | -2,3981 | 0,0019 | 0,0068 | 0,0032 | 4363 | tags=69%, list=23%, signal=53% | 1457/55781/25996/84128/29107/83732/4931/10171/55131/29889/10248/84135/10813/23195/102157402/3692/6949/55272/51077/55127/10199/92856/23560/84916/55916/2091/134430/6023/55226/5901/65083/54552/4809/56000/23160/26354/51367/10436/166378/51602/81691/27341/1736/10885/5822/9790/10940/54433/55651/10799/10528/54913 |
| **hsa00062** | Fatty acid elongation | 27 | -0,6332 | -1,9588 | 0,0020 | 0,0068 | 0,0032 | 3251 | tags=48%, list=17%, signal=40% | 9200/54898/9374/401494/1892/79071/117145/83401/51495/51102/3033/11332 |
| **hsa03420** | Nucleotide excision repair | 46 | -0,6324 | -2,1977 | 0,0019 | 0,0068 | 0,0032 | 3833 | tags=52%, list=20%, signal=42% | 6118/4331/902/5981/5424/728340/6117/10714/2967/2966/1069/54107/2068/5982/5984/5426/5985/5111/6119/5983/5425/3978/5427 |
| **hsa03020** | RNA polymerase | 28 | -0,6311 | -1,9768 | 0,0019 | 0,0068 | 0,0032 | 5382 | tags=71%, list=28%, signal=51% | 10623/30834/10621/5441/5432/5437/64425/5436/11128/5440/9533/84172/5438/171568/221830/5433/51728/10622/55703 |
| **hsa00970** | Aminoacyl-tRNA biosynthesis | 25 | -0,6241 | -1,9168 | 0,0020 | 0,0068 | 0,0032 | 6325 | tags=88%, list=33%, signal=59% | 55699/57038/92935/283459/55278/10352/51091/51067/10667/25973/54938/79731/23395/123263/57505/5188/55157/79587/124454/2193/10056 |
| **hsa00650** | Butanoate metabolism | 23 | -0,6052 | -1,8186 | 0,0020 | 0,0068 | 0,0032 | 2514 | tags=52%, list=13%, signal=45% | 6296/56898/1962/1892/38/622/79944/3033/64064/7915/5019 |
| **hsa03050** | Proteasome | 45 | -0,6035 | -2,0859 | 0,0019 | 0,0068 | 0,0032 | 6234 | tags=73%, list=33%, signal=49% | 5706/5717/5700/5714/5692/5688/5689/5691/5699/5701/5693/5713/9861/5686/51371/5705/5682/10213/5719/5698/5690/5720/5707/5684/5696/5718/5683/5685/5702/10197/5721/143471 |
| **hsa05144** | Malaria | 48 | 0,5981 | 2,1028 | 0,0020 | 0,0068 | 0,0032 | 3415 | tags=52%, list=18%, signal=43% | 4233/54106/3576/3553/6383/3586/3039/4035/3043/6382/3040/2532/6347/1379/7412/2994/2993/7124/7043/3820/7060/7097/7057/958/22914 |
| **hsa04110** | Cell cycle | 123 | -0,5943 | -2,4409 | 0,0020 | 0,0068 | 0,0032 | 2756 | tags=43%, list=15%, signal=37% | 23595/9232/51433/8243/891/3066/894/9700/9133/1031/1870/64682/5001/11200/7027/1017/896/991/7029/7157/699/995/8317/5591/1021/4174/7272/701/5111/890/983/5933/1019/7465/6502/4609/23594/4172/9134/4998/4171/4176/4085/898/4173/9088/4175/1111/1869/993/990/8318 |
| **hsa01040** | Biosynthesis of unsaturated fatty acids | 27 | -0,5842 | -1,8073 | 0,0059 | 0,0163 | 0,0075 | 4183 | tags=63%, list=22%, signal=49% | 3992/8310/201562/51/570/9524/51144/9200/9415/54898/401494/79071/83401/79966/51495/11332 |
| **hsa05332** | Graft-versus-host disease | 36 | 0,5808 | 1,9414 | 0,0041 | 0,0121 | 0,0056 | 6729 | tags=75%, list=35%, signal=49% | 3002/3553/940/356/3552/3821/7124/3133/3117/5551/3134/3811/3119/3558/3824/3106/3135/3812/3108/3804/3802/3122/3109/3127/3107/3115/3112 |
| **hsa01212** | Fatty acid metabolism | 56 | -0,5716 | -2,0568 | 0,0019 | 0,0068 | 0,0032 | 3251 | tags=45%, list=17%, signal=37% | 1374/36/9200/9415/39/31/54898/84869/1376/34/9374/1962/2194/401494/27349/1892/38/79071/83401/79966/7923/51495/51102/3033 |
| **hsa00630** | Glyoxylate and dicarboxylate metabolism | 28 | -0,5702 | -1,7861 | 0,0058 | 0,0163 | 0,0075 | 5120 | tags=68%, list=27%, signal=50% | 283871/48/9380/84693/5096/1431/4190/1738/4191/84532/39/6472/847/38/6470/125061/2653/189 |
| **hsa05323** | Rheumatoid arthritis | 86 | 0,5689 | 2,2379 | 0,0021 | 0,0068 | 0,0032 | 2596 | tags=36%, list=14%, signal=31% | 4312/1514/3725/6364/7422/534/2920/3576/6349/2921/3553/940/3552/245972/1493/6348/1435/6347/54/9296/1513/535/7124/2321/10312/537/3117/6352/7043/1437/3600 |
| **hsa00240** | Pyrimidine metabolism | 56 | -0,5631 | -2,0263 | 0,0019 | 0,0068 | 0,0032 | 2127 | tags=38%, list=11%, signal=33% | 654364/56953/29922/790/56474/115024/7372/7371/1503/1723/79077/6240/4833/1841/4830/5169/1854/6241/7083/7298 |
| **hsa00280** | Valine, leucine and isoleucine degradation | 45 | -0,5602 | -1,9361 | 0,0019 | 0,0068 | 0,0032 | 4607 | tags=62%, list=24%, signal=47% | 10449/1629/84693/5096/4329/1738/36/56922/11112/3028/39/3712/26275/34/217/1962/587/64087/1892/38/594/586/3033/64064/5019/219/501 |
| **hsa00563** | Glycosylphosphatidylinositol (GPI)-anchor biosynthesis | 24 | -0,5593 | -1,6924 | 0,0081 | 0,0204 | 0,0094 | 5322 | tags=58%, list=28%, signal=42% | 80235/23556/2822/51604/5281/9488/51227/93183/8733/128869/10026/54965/284098 |
| **hsa04710** | Circadian rhythm | 30 | 0,5538 | 1,7573 | 0,0084 | 0,0209 | 0,0096 | 5122 | tags=60%, list=27%, signal=44% | 5187/53632/4862/8553/6095/9572/1453/1454/79365/1407/51422/1408/5565/8863/6097/5564/23291/406 |
| **hsa05143** | African trypanosomiasis | 36 | 0,5507 | 1,8408 | 0,0062 | 0,0163 | 0,0075 | 2808 | tags=36%, list=15%, signal=31% | 54106/3553/356/335/3586/3039/3043/3040/7412/5582/7124/3620/5579 |
| **hsa00020** | Citrate cycle (TCA cycle) | 29 | -0,5507 | -1,7487 | 0,0038 | 0,0118 | 0,0055 | 5506 | tags=69%, list=29%, signal=49% | 8803/47/6392/5162/6391/48/8802/8801/1431/6390/4190/1738/4191/5160/3419/5106/3418/1737/2271 |
| **hsa00900** | Terpenoid backbone biosynthesis | 21 | -0,5492 | -1,6158 | 0,0154 | 0,0335 | 0,0154 | 2845 | tags=38%, list=15%, signal=32% | 2224/39/10269/23463/10654/23590/38 |
| **hsa03060** | Protein export | 23 | -0,5482 | -1,6473 | 0,0120 | 0,0273 | 0,0126 | 6431 | tags=78%, list=34%, signal=52% | 6729/29927/10952/90701/11231/6728/6727/6731/23480/60559/6734/196294/3309/28972/83943/6726/58477 |
| **hsa00270** | Cysteine and methionine metabolism | 46 | -0,5459 | -1,8971 | 0,0019 | 0,0068 | 0,0032 | 4447 | tags=52%, list=23%, signal=40% | 6898/58478/7263/3939/1789/4190/2805/4191/55256/875/113675/262/84245/587/4507/191/3945/160287/2806/586/6723/29968/26227 |
| **hsa00983** | Drug metabolism - other enzymes | 59 | -0,5429 | -1,9647 | 0,0019 | 0,0068 | 0,0032 | 2387 | tags=37%, list=13%, signal=33% | 3615/8824/3614/8833/654364/3704/2950/29922/7372/1571/3251/7371/2941/6240/4258/4833/4830/1854/6241/7083/4353 |
| **hsa05320** | Autoimmune thyroid disease | 39 | 0,5426 | 1,8342 | 0,0041 | 0,0121 | 0,0056 | 5257 | tags=49%, list=28%, signal=35% | 7038/3002/940/356/3586/1493/3133/3117/5551/3134/958/3119/3558/7253/959/3106/3135/7173/3108 |
| **hsa04640** | Hematopoietic cell lineage | 90 | 0,5365 | 2,1298 | 0,0021 | 0,0068 | 0,0032 | 2677 | tags=41%, list=14%, signal=35% | 4311/3690/3554/3553/3563/4254/3552/3675/921/928/914/916/7850/1435/3672/915/925/1379/2814/926/3684/1438/909/960/1436/3566/2993/7124/911/3117/917/2209/1437/2057/931/3575/3673 |
| **hsa00260** | Glycine, serine and threonine metabolism | 39 | -0,5275 | -1,7781 | 0,0019 | 0,0068 | 0,0032 | 2421 | tags=44%, list=13%, signal=38% | 63826/1757/875/113675/5223/6472/2628/5723/6470/23464/29968/2653/189/501/26227/2593 |
| **hsa00340** | Histidine metabolism | 23 | -0,5229 | -1,5712 | 0,0200 | 0,0403 | 0,0186 | 3271 | tags=43%, list=17%, signal=36% | 220/26/138199/217/144193/3067/219/3034/501 |
| **hsa03010** | Ribosome | 130 | -0,5204 | -2,1600 | 0,0019 | 0,0068 | 0,0032 | 7342 | tags=71%, list=39%, signal=44% | 6138/6187/6124/4736/6227/6218/6188/6159/6165/6164/6132/9349/6142/25873/6223/6230/2197/6168/6157/6147/64981/6152/6155/6194/65005/6125/6201/6133/6154/6206/6130/6228/6229/6204/6166/64928/29093/6144/64983/6173/6129/6233/6189/6146/6139/6175/6193/9045/11224/6231/63931/55168/51116/6150/9553/64960/10573/51264/51187/6128/54460/54948/51023/3921/64963/29088/51069/51263/55173/64965/51073/51121/29074/51021/51318/9801/64979/51373/6183/219927/11222/28998/6123/55052/51081/65003/200916/79590/65008/63875/6182 |
| **hsa00640** | Propanoate metabolism | 32 | -0,5198 | -1,6871 | 0,0038 | 0,0118 | 0,0055 | 6005 | tags=78%, list=32%, signal=54% | 8310/51/8803/55862/593/1629/8802/8801/84693/5096/4329/3939/1738/32/84532/39/31/26275/1962/1892/38/594/3945/160287 |
| **hsa04929** | GnRH secretion | 61 | 0,5189 | 1,9033 | 0,0021 | 0,0068 | 0,0032 | 3052 | tags=36%, list=16%, signal=30% | 6696/3762/2100/3780/610/3763/3782/3767/3710/5582/2550/778/2796/3708/10000/3783/5579/7220/2797/409/8913/5604 |
| **hsa04380** | Osteoclast differentiation | 123 | 0,5133 | 2,1489 | 0,0020 | 0,0068 | 0,0032 | 4450 | tags=48%, list=23%, signal=37% | 3725/3690/3554/5468/3553/2212/4688/5603/23547/3552/4286/814/2355/1435/9846/4792/4791/3932/54/11025/9021/1513/1436/8878/23118/2534/126014/2274/3726/7124/11027/3459/5533/11006/2209/10859/10000/5971/79168/5604/7046/2354/7048/140885/2213/8517/9020/54209/29760/10379/6688/3727/5609/3460/7189/55423/3937/7297/5970 |
| **hsa04940** | Type I diabetes mellitus | 38 | 0,5130 | 1,7209 | 0,0063 | 0,0163 | 0,0075 | 3227 | tags=37%, list=17%, signal=31% | 2571/3002/3553/940/356/4049/3552/7124/3133/3117/5551/3134/3119/3558 |
| **hsa04330** | Notch signaling pathway | 53 | 0,5127 | 1,8237 | 0,0021 | 0,0068 | 0,0032 | 4443 | tags=49%, list=23%, signal=38% | 4854/28514/3280/1840/3714/6310/84441/5986/1857/23220/1387/4851/342371/8650/196403/1855/9612/83464/6868/4853/2033/1856/9794/388585/113878/3516 |
| **hsa04061** | Viral protein interaction with cytokine and cytokine receptor | 92 | 0,5118 | 2,0441 | 0,0020 | 0,0068 | 0,0032 | 2681 | tags=41%, list=14%, signal=36% | 6354/643/6364/29949/2920/3576/6349/2921/11009/7133/2829/8793/3586/4049/8740/6375/2833/1230/6348/1435/3560/6347/1236/8764/1232/2826/1436/6846/6369/1233/6362/7124/10803/6361/3572/6352/3588/8807 |
| **hsa00071** | Fatty acid degradation | 41 | -0,5111 | -1,7498 | 0,0019 | 0,0068 | 0,0032 | 4604 | tags=49%, list=24%, signal=37% | 10449/128/10455/130/125/1374/36/1632/39/1376/34/217/1962/1892/38/2639/3033/219/501 |
| **hsa05217** | Basal cell carcinoma | 61 | 0,5090 | 1,8669 | 0,0021 | 0,0068 | 0,0032 | 3949 | tags=44%, list=21%, signal=35% | 7474/2737/7477/1026/81029/8322/10912/51176/5727/7482/6932/7483/83439/1857/7476/80326/324/8312/10297/64399/1855/1499/652/51426/1856/8325/7855 |
| **hsa04512** | ECM-receptor interaction | 83 | 0,5075 | 1,9693 | 0,0021 | 0,0068 | 0,0032 | 3091 | tags=41%, list=16%, signal=34% | 6696/3696/3909/22987/3914/3690/1284/22801/22798/3675/3908/2335/3915/3672/3685/1292/6382/1291/2814/256076/960/7143/7148/1286/7450/5649/3918/3688/3694/3673/7060/3693/1605/7057 |
| **hsa05330** | Allograft rejection | 34 | 0,5058 | 1,6629 | 0,0062 | 0,0163 | 0,0075 | 4229 | tags=41%, list=22%, signal=32% | 3002/940/356/3586/7124/3133/3117/5551/3134/958/3119/3558/959/3106 |
| **hsa00480** | Glutathione metabolism | 48 | -0,5054 | -1,7609 | 0,0039 | 0,0118 | 0,0055 | 2387 | tags=31%, list=13%, signal=27% | 27306/3418/79017/2950/4953/51471/79094/2941/6240/6723/4258/494143/2882/6241 |
| **hsa04966** | Collecting duct acid secretion | 26 | 0,5054 | 1,5658 | 0,0141 | 0,0313 | 0,0144 | 4755 | tags=58%, list=25%, signal=43% | 534/245972/6521/9296/495/535/10312/760/525/526/528/9550/523/527/50617 |
| **hsa04064** | NF-kappa B signaling pathway | 98 | 0,5033 | 2,0384 | 0,0020 | 0,0068 | 0,0032 | 5011 | tags=54%, list=26%, signal=40% | 3554/2920/3576/2921/3553/27040/597/4049/8740/29775/5328/79092/84433/7128/4792/4791/7185/3932/7412/10892/23118/353376/7124/23643/598/148022/329/5971/5579/5743/9560/958/7535/330/10913/8517/9020/5335/29760/8717/959/472/51588/7189/60401/6351/5970/3383/6363/4055/128178/4067/3551 |
| **hsa05142** | Chagas disease (American trypanosomiasis) | 99 | 0,5001 | 2,0255 | 0,0020 | 0,0068 | 0,0032 | 3534 | tags=37%, list=19%, signal=31% | 3725/54106/2775/3576/6349/5054/3553/5603/356/3586/916/6348/915/919/4792/10333/6347/7124/3459/6352/7043/917/10000/148022/5516/7097/7046/7048/2774/3558/9630/713/718/55844/8517/5521/4087 |
| **hsa01523** | Antifolate resistance | 30 | -0,4978 | -1,5906 | 0,0190 | 0,0387 | 0,0179 | 1711 | tags=33%, list=9%, signal=30% | 471/2618/113235/6573/6470/2352/8836/1719/7298 |
| **hsa00380** | Tryptophan metabolism | 42 | -0,4953 | -1,6978 | 0,0057 | 0,0163 | 0,0075 | 2960 | tags=40%, list=16%, signal=34% | 26/55526/39/23498/217/1962/51166/847/1892/38/2639/1644/3033/125061/219/501 |
| **hsa05150** | Staphylococcus aureus infection | 68 | 0,4944 | 1,8566 | 0,0021 | 0,0068 | 0,0032 | 3524 | tags=40%, list=19%, signal=32% | 3866/3868/8689/728/2212/3586/2204/3684/10747/3872/1668/715/3117/721/2209/1669/2359/2357/820/3119/2213/713/718/720/5724/719/3860 |
| **hsa01230** | Biosynthesis of amino acids | 69 | -0,4942 | -1,8426 | 0,0019 | 0,0068 | 0,0032 | 2432 | tags=33%, list=13%, signal=29% | 875/113675/1373/3419/5223/5631/3418/6472/587/5634/100526760/2806/586/5723/6470/5832/29968/95/84706/5831/26227/65263 |
| **hsa03013** | RNA transport | 165 | -0,4938 | -2,0995 | 0,0019 | 0,0068 | 0,0032 | 5392 | tags=55%, list=28%, signal=39% | 7514/10284/51095/59343/6612/79760/26019/8890/728689/7329/11171/6613/3646/1981/348995/10556/10921/1967/8668/9086/8086/53371/11218/29107/4116/79902/1979/9775/51808/26835/1977/23165/8669/10248/11260/8663/8480/8662/96764/8894/7341/22916/81929/5905/23279/9939/57122/1965/8487/26834/11102/79833/9688/23511/8637/55916/3837/60528/4927/5901/80145/65109/57510/10460/56000/4686/25929/101954264/84321/1964/129401/55520/8891/10419/6606/79023/51367/1207/10189/9669/55706/50628/10940/9631/1978/10799/54913/23225/55110 |
| **hsa05211** | Renal cell carcinoma | 68 | 0,4931 | 1,8516 | 0,0021 | 0,0068 | 0,0032 | 3340 | tags=31%, list=18%, signal=26% | 4233/3725/9915/7422/2889/3091/1026/2034/1387/201163/673/7030/54583/2113/7043/10000/5155/6513/5604/1398/2033 |
| **hsa04660** | T cell receptor signaling pathway | 101 | 0,4929 | 2,0000 | 0,0020 | 0,0068 | 0,0032 | 5078 | tags=46%, list=27%, signal=34% | 3725/5133/27040/5603/940/3586/1493/916/8440/915/925/919/84433/4792/3932/926/10892/10125/2534/29851/7124/868/3702/1326/5533/9402/917/1437/10000/5604/3558/7535/8517/10451/9020/5335/959/5609/3937/6654/5970/5777/2932/4773/3551/5788 |
| **hsa05120** | Epithelial cell signaling in Helicobacter pylori infection | 68 | 0,4921 | 1,8480 | 0,0021 | 0,0068 | 0,0032 | 5397 | tags=56%, list=28%, signal=40% | 4233/3725/534/2920/3576/2921/5603/6714/245972/4792/1839/58494/9296/535/10312/537/6352/7082/6868/51606/8517/9020/5335/102/525/526/528/9550/5970/523/527/50617/50848/4067/3551/2919/51382/3577 |
| **hsa04976** | Bile secretion | 58 | 0,4891 | 1,7857 | 0,0021 | 0,0068 | 0,0032 | 3160 | tags=45%, list=17%, signal=37% | 114/8671/9429/6580/6550/366/1244/196883/358/5243/343/57552/6343/112/8714/486/1576/1581/115/2052/6513/6522/482/760/113/8431 |
| **hsa05412** | Arrhythmogenic right ventricular cardiomyopathy (ARVC) | 72 | 0,4877 | 1,8623 | 0,0021 | 0,0068 | 0,0032 | 3328 | tags=40%, list=18%, signal=33% | 3696/59283/1756/3690/89/9254/22801/3675/3908/88/6445/3672/3685/51176/6932/83439/6262/59285/778/3688/3694/3673/6543/4000/1495/3693/1605/5318/1499 |
| **hsa05216** | Thyroid cancer | 36 | 0,4877 | 1,6304 | 0,0103 | 0,0242 | 0,0112 | 4500 | tags=47%, list=24%, signal=36% | 5468/7849/1026/10912/5979/51176/6932/83439/999/673/595/5604/1499/51426/6256/6257/1647 |
| **hsa04916** | Melanogenesis | 94 | 0,4857 | 1,9553 | 0,0020 | 0,0068 | 0,0032 | 4281 | tags=43%, list=23%, signal=33% | 7474/2775/7477/114/4254/4286/81029/8322/196883/51176/7482/6932/5582/7483/83439/1857/7476/1387/817/80326/112/4157/115/1906/1855/5579/1910/434/5604/113/1499/2033/111/5443/1856/8325/7855/816/815/5331 |
| **hsa04668** | TNF signaling pathway | 111 | 0,4844 | 1,9868 | 0,0020 | 0,0068 | 0,0032 | 3052 | tags=32%, list=16%, signal=27% | 3725/6364/3976/2277/2920/2921/1051/7133/3553/5603/7424/4049/4318/1435/7128/4792/4323/7185/6347/7412/9021/153090/23118/3726/7124/602/9586/1326/6352/1437/10000/3600/1906/329/5743/5604 |
| **hsa05012** | Parkinson disease | 122 | -0,4831 | -1,9825 | 0,0020 | 0,0068 | 0,0032 | 5201 | tags=57%, list=27%, signal=41% | 51079/1337/9246/4541/4512/2773/7326/4728/7416/6392/4719/4704/4513/4519/5567/4723/4731/1537/6391/4716/55967/54539/27429/29796/4725/4713/7318/6390/4722/4537/6570/7332/4697/7388/4717/10975/4538/6571/4702/4540/4696/4711/1329/1349/9377/4709/4701/126328/4715/1345/4714/4712/4707/11315/4698/4706/4726/4514/7419/4536/4535/4508/4539/4718/27089/4509/54205/56901 |
| **hsa00330** | Arginine and proline metabolism | 49 | -0,4823 | -1,6870 | 0,0058 | 0,0163 | 0,0075 | 1187 | tags=27%, list=6%, signal=25% | 4953/2806/5832/6723/79814/219/5625/5831/102724788/501/65263/2593 |
| **hsa04510** | Focal adhesion | 193 | 0,4797 | 2,1079 | 0,0020 | 0,0068 | 0,0032 | 3538 | tags=36%, list=19%, signal=30% | 4233/6696/53358/5923/3696/3909/5154/3725/3914/3690/7422/2277/2318/1284/2889/6714/22801/5159/7424/22798/3675/3908/824/2335/3915/3672/5747/3685/1292/1291/9564/5582/256076/857/2534/7143/7148/23396/1286/673/5228/7450/1950/595/2321/394/5649/25759/3611/2316/10000/3918/5155/3688/3694/3673/329/5579/7060/3693/7423/5604/7057/1398/1499/330/3480/10451/54776/1280 |
| **hsa03040** | Spliceosome | 144 | -0,4790 | -2,0188 | 0,0019 | 0,0068 | 0,0032 | 4276 | tags=44%, list=23%, signal=35% | 9879/6626/10915/10450/10262/9343/144983/4116/10907/494115/9775/10286/26835/6633/51639/153527/6628/10713/11157/3192/26121/51690/9128/6629/27316/23350/22916/9939/84844/26834/10772/23450/27258/9410/6631/6434/6634/83443/6637/6627/10465/10929/4809/220988/10946/27339/4686/6428/101954264/84321/25804/6432/6636/6635/3178/10189/4670/6427/6426/23658/6632/51645/55110 |
| **hsa00600** | Sphingolipid metabolism | 45 | 0,4721 | 1,6285 | 0,0063 | 0,0163 | 0,0075 | 5088 | tags=47%, list=27%, signal=34% | 129807/9514/8613/56624/8877/81537/7357/427/8612/204219/4758/57704/259230/7368/10825/8879/55512/64781/79603/29956/2720 |
| **hsa05205** | Proteoglycans in cancer | 196 | 0,4706 | 2,0732 | 0,0021 | 0,0068 | 0,0032 | 3639 | tags=36%, list=19%, signal=29% | 4233/7474/2017/2247/1514/3690/7422/7477/2318/5329/5603/6714/6383/356/7074/3091/4318/1026/5328/81029/2335/8322/23365/5747/3685/6382/5727/7482/22808/3710/1839/5582/7483/857/286/2065/960/7476/7078/817/22800/673/80326/7124/595/2817/288/8826/6774/3708/2316/10000/3688/3673/5579/5962/3693/7097/5604/867/7057/1499/3480/10451/54776/10855/4087/967/5335/3316 |
| **hsa05235** | PD-L1 expression and PD-1 checkpoint pathway in cancer | 89 | 0,4677 | 1,8556 | 0,0020 | 0,0068 | 0,0032 | 3611 | tags=33%, list=19%, signal=27% | 3725/54106/5133/27040/5603/940/3091/238/916/915/919/4792/3932/10125/29126/353376/1950/3459/116071/6774/5533/917/10000/148022/7097/5604/7535/8517/5335 |
| **hsa04911** | Insulin secretion | 72 | 0,4664 | 1,7810 | 0,0021 | 0,0068 | 0,0032 | 3156 | tags=35%, list=17%, signal=29% | 1131/6804/114/3780/196883/3782/3767/3710/2740/5582/11069/6262/817/3778/778/112/6844/486/9586/115/3783/5579/6513/482/113 |
| **hsa04146** | Peroxisome | 80 | -0,4636 | -1,7529 | 0,0020 | 0,0068 | 0,0032 | 6159 | tags=60%, list=32%, signal=41% | 5052/6342/23417/8443/8310/373156/4358/9409/51/5824/10005/5193/1384/5830/6647/51703/84188/54677/26061/283927/570/1891/10455/25824/8540/11264/8528/10901/83594/5825/8504/23600/3418/5264/1962/5192/847/10654/92960/5191/10478/8309/5827/26063/11001/189/255027 |
| **hsa00620** | Pyruvate metabolism | 38 | -0,4630 | -1,5573 | 0,0229 | 0,0450 | 0,0208 | 3675 | tags=53%, list=19%, signal=43% | 4190/1738/32/4191/197257/84532/5160/39/31/5106/217/1737/2739/38/2271/3945/160287/219/501 |
| **hsa05140** | Leishmaniasis | 71 | 0,4615 | 1,7608 | 0,0021 | 0,0068 | 0,0032 | 3317 | tags=32%, list=17%, signal=27% | 3725/3553/2212/4688/5603/3586/3552/4792/1379/3684/23118/7124/1917/3459/3117/7043/2209/3688/5579/5743/7097/3119/718 |
| **hsa05100** | Bacterial invasion of epithelial cells | 64 | 0,4598 | 1,7045 | 0,0021 | 0,0068 | 0,0032 | 3328 | tags=31%, list=18%, signal=26% | 4233/53358/2017/6714/2335/5747/9564/79658/857/999/8976/25759/3611/3688/1495/867/79767/1398/26052/1499 |
| **hsa05134** | Legionellosis | 57 | 0,4596 | 1,6707 | 0,0062 | 0,0163 | 0,0075 | 2256 | tags=28%, list=12%, signal=25% | 2920/3576/2921/3553/4792/7100/4791/1379/3304/3684/3310/1195/3303/7124/1917/664 |
| **hsa04933** | AGE-RAGE signaling pathway in diabetic complications | 98 | 0,4590 | 1,8591 | 0,0020 | 0,0068 | 0,0032 | 3611 | tags=33%, list=19%, signal=27% | 7056/3725/7422/2277/3576/1284/5054/3553/5603/7424/3552/2335/6347/7412/4088/1286/2308/5292/7124/595/2152/6774/7043/10000/1906/5579/7423/7046/7048/4087/5581/5335 |
| **hsa05032** | Morphine addiction | 75 | 0,4560 | 1,7462 | 0,0021 | 0,0068 | 0,0032 | 3242 | tags=31%, list=17%, signal=26% | 5138/2791/5141/3762/2775/114/5139/3763/196883/5582/2550/5143/5150/112/2785/115/54331/50940/5579/409/774/113/2570 |
| **hsa04520** | Adherens junction | 70 | 0,4558 | 1,7220 | 0,0021 | 0,0068 | 0,0032 | 3662 | tags=36%, list=19%, signal=29% | 4233/10810/81607/6714/10458/51176/6932/83439/999/1387/2534/4088/8976/8826/5787/7082/4301/1495/7046/7048/1499/2033/25945/3480/51701 |
| **hsa05321** | Inflammatory bowel disease (IBD) | 60 | 0,4540 | 1,6684 | 0,0021 | 0,0068 | 0,0032 | 5734 | tags=55%, list=30%, signal=39% | 3725/3553/3586/3552/7100/6775/2625/3566/4088/3596/7124/30009/3459/6095/3117/6774/7043/8807/7097/3119/3558/4087/4094/3460/5970/149233/8809/6097/3108/7040/4772/4790/3122 |
| **hsa05410** | Hypertrophic cardiomyopathy (HCM) | 84 | 0,4470 | 1,7413 | 0,0021 | 0,0068 | 0,0032 | 3750 | tags=38%, list=20%, signal=31% | 3696/59283/1756/3690/9254/22801/3675/3908/53632/7168/6445/3672/3685/6262/59285/778/7124/7043/3688/1906/3694/3673/7169/6543/4000/3693/1605/7137/51422/4607/8516/5565 |
| **hsa04658** | Th1 and Th2 cell differentiation | 88 | 0,4408 | 1,7385 | 0,0021 | 0,0068 | 0,0032 | 4450 | tags=41%, list=23%, signal=31% | 4854/28514/3725/27040/5603/3714/916/84441/915/919/864/4792/3560/6775/3932/2625/3566/3596/4851/30009/3459/3117/5533/917/3119/3558/4853/7535/8517/5335/4094/3460/9794/7297/3516/5970 |
| **hsa05414** | Dilated cardiomyopathy (DCM) | 88 | 0,4406 | 1,7374 | 0,0021 | 0,0068 | 0,0032 | 3156 | tags=35%, list=17%, signal=30% | 3696/59283/1756/3690/114/9254/22801/3675/3908/7168/6445/3672/3685/196883/6262/59285/778/112/7124/115/7043/3688/3694/3673/7169/6543/4000/3693/1605/7137/113 |
| **hsa04015** | Rap1 signaling pathway | 197 | 0,4404 | 1,9405 | 0,0021 | 0,0068 | 0,0032 | 3714 | tags=37%, list=20%, signal=30% | 4233/5154/2247/3690/7422/2775/2277/114/2889/27040/9732/1946/5603/6714/4254/5159/7074/7424/5909/135/8631/1435/2254/196883/9564/22808/57568/5582/3684/8822/11069/999/1436/5900/673/5228/112/1950/2321/1268/5898/25780/2904/9771/115/64411/26037/10000/5155/3688/9693/51466/5579/4301/9170/83593/7423/5604/2357/7057/113/1398/9223/51378/1499/3480/2246/10451/111/5335/25865/1399 |
| **hsa04115** | p53 signaling pathway | 70 | -0,4399 | -1,6443 | 0,0057 | 0,0163 | 0,0075 | 2247 | tags=30%, list=12%, signal=27% | 5366/894/9133/11200/1017/896/9538/7157/10572/51512/54205/1021/55240/983/1019/9134/898/6241/1111/7161 |
| **hsa04620** | Toll-like receptor signaling pathway | 90 | 0,4398 | 1,7462 | 0,0021 | 0,0068 | 0,0032 | 3144 | tags=29%, list=17%, signal=24% | 6696/3725/54106/3576/6349/3553/5603/6348/4792/7100/10333/3663/1513/23118/353376/3665/7124/23643/1326/6352/10000/148022/7097/5604/9560/958 |
| **hsa05145** | Toxoplasmosis | 107 | 0,4396 | 1,7934 | 0,0021 | 0,0068 | 0,0032 | 4450 | tags=41%, list=23%, signal=32% | 3909/3914/2775/5603/3586/22798/3908/3915/4792/23533/3304/3310/23118/3303/7124/3459/23643/3117/6774/598/7043/10000/3918/3688/3588/329/7097/958/3119/146850/330/1234/8517/4261/3587/3305/3913/959/3912/284217/3460/7189/7297/5970 |
| **hsa04727** | GABAergic synapse | 72 | 0,4392 | 1,6769 | 0,0021 | 0,0068 | 0,0032 | 3550 | tags=36%, list=19%, signal=29% | 2791/2571/2775/114/6714/3763/196883/5582/18/2550/2752/778/112/5334/2785/6539/115/27165/23710/54331/5579/774/113/2570/6540/111 |
| **hsa05418** | Fluid shear stress and atherosclerosis | 129 | 0,4366 | 1,8337 | 0,0020 | 0,0068 | 0,0032 | 2616 | tags=28%, list=14%, signal=24% | 7056/5154/1514/3725/3690/7422/3554/3553/4688/5603/90/6714/6383/10365/3552/1843/4318/7850/5747/3685/6382/6347/7412/659/857/5327/119391/8878/7124/2817/387082/2949/657/10000/5155/1906 |
| **hsa04659** | Th17 cell differentiation | 100 | 0,4358 | 1,7701 | 0,0020 | 0,0068 | 0,0032 | 4521 | tags=39%, list=24%, signal=30% | 3725/3554/3553/27040/5603/3091/916/915/919/4792/3560/3932/2625/3566/4088/30009/3459/6095/3117/6774/5533/3572/917/5914/7046/7048/3119/3558/7535/8517/4087/5335/6256/3662/6257/3460/7297/5970/149233 |
| **hsa05135** | Yersinia infection | 118 | 0,4314 | 1,7973 | 0,0020 | 0,0068 | 0,0032 | 5669 | tags=47%, list=30%, signal=33% | 3725/3576/6196/3553/27040/5603/6714/3586/147945/2335/10458/23365/5747/4792/9564/6347/3932/23118/23396/7456/7124/8976/3984/10000/3688/148022/5604/1398/3558/7535/8517/10451/382/5335/1399/5609/7189/3937/5970/147179/5829/9138/2932/4773/2533/8874/3551/10163/3678/114548/4772/6197/4790/6195/7409 |
| **hsa04928** | Parathyroid hormone synthesis, secretion and action | 98 | 0,4314 | 1,7472 | 0,0020 | 0,0068 | 0,0032 | 3962 | tags=39%, list=21%, signal=31% | 5141/632/9935/114/8521/1026/9826/196883/4323/3710/1839/5582/2625/4929/5338/5143/10672/673/112/9586/7421/142680/3708/115/5337/860/2768/5579/409/5604/113/9365/6667/111/11214/10893/6256/3727 |
| **hsa04060** | Cytokine-cytokine receptor interaction | 256 | 0,4297 | 1,9499 | 0,0021 | 0,0068 | 0,0032 | 3561 | tags=36%, list=19%, signal=30% | 6354/9966/8808/643/6364/3976/3554/130399/29949/2920/3576/6349/2921/11009/7133/3553/2829/163702/90/10663/3563/8742/356/8793/58191/3586/4049/8740/9180/3552/6375/9518/27302/3953/2833/7850/1230/6348/1435/3557/3560/2658/6347/1236/659/27177/1438/8764/1232/2826/1436/6846/2661/3566/6369/1233/6362/3596/7124/3459/10803/8784/23495/133396/6361/3572/6352/246778/7043/657/1437/8744/3600/2057/3575/3588/8807/91/3603/7046/7048/9560/958/51330/3558/1234/51554/10913/652/939/93/9235 |
| **hsa04666** | Fc gamma R-mediated phagocytosis | 91 | 0,4278 | 1,7080 | 0,0020 | 0,0068 | 0,0032 | 3714 | tags=33%, list=20%, signal=27% | 10810/8613/85477/27040/2212/8877/50807/274/9846/5582/4082/8612/8605/5338/23396/5337/3984/2209/10000/8398/5579/5604/1398/2213/2934/10451/382/5581/5335/1399 |
| **hsa04010** | MAPK signaling pathway | 276 | 0,4274 | 1,9530 | 0,0021 | 0,0068 | 0,0032 | 4622 | tags=43%, list=24%, signal=33% | 4233/1850/5923/5154/59283/2247/3725/2069/7422/3554/2277/374/2318/6196/3553/9254/1946/5603/4254/5159/356/1846/1847/7424/3552/1843/1848/1435/10912/2254/23162/4791/80824/22808/5582/3304/10125/3310/8822/2065/1436/23118/7786/22800/8605/11221/59285/23542/778/673/3164/5228/3303/7124/1950/2321/5801/8569/5921/25780/1326/5533/7043/2768/2316/10000/5155/9693/5971/5579/8491/5495/7423/409/8913/774/10746/5604/7046/7048/115727/1398/51776/6788/51378/5924/627/3480/9448/2246/8517/1649/5494/9020/51701/1399/8717/9479/8911/3305/3727/4763/57551/777/5609/2253/7189/7010/9261/1845/6654/4215/2122/5970/1969/1647/1386/8912/2872 |
| **hsa04610** | Complement and coagulation cascades | 74 | 0,4248 | 1,6277 | 0,0021 | 0,0068 | 0,0032 | 3530 | tags=35%, list=19%, signal=29% | 7056/3687/5055/728/5054/5329/2/2159/5328/1379/3684/5327/10747/7035/715/5345/7450/2152/721/735/462/713/718/720/719/3053 |
| **hsa04721** | Synaptic vesicle cycle | 70 | 0,4236 | 1,6004 | 0,0021 | 0,0068 | 0,0032 | 4755 | tags=46%, list=25%, signal=34% | 6506/6804/534/245972/6857/9296/535/6844/10312/6539/112755/6812/774/26052/6809/51606/6540/2054/6505/6536/57030/6507/161/525/526/528/1785/9550/523/527/6511/50617 |
| **hsa01200** | Carbon metabolism | 109 | -0,4206 | -1,6882 | 0,0039 | 0,0118 | 0,0055 | 4553 | tags=43%, list=24%, signal=33% | 6391/48/8802/128/8801/2098/84693/5096/1431/4329/6390/2023/25796/4190/1738/2805/4191/414328/26007/84532/5160/39/22934/113675/1373/3419/26275/5223/5631/3418/1737/6472/1962/5634/847/1892/38/2271/2806/5723/6470/29968/2653/189/84706/26227 |
| **hsa04014** | Ras signaling pathway | 209 | 0,4172 | 1,8576 | 0,0021 | 0,0068 | 0,0032 | 3497 | tags=32%, list=18%, signal=26% | 4233/53358/2791/5923/5154/2247/7422/2277/27040/1946/4254/5159/356/7074/7424/9462/8437/1435/9846/2254/2114/22808/5582/10125/8822/3363/1436/22800/8605/5338/5900/5228/1950/2321/2785/2113/25759/5898/5921/23179/25780/2904/9771/5337/598/10000/5155/54331/5863/8398/5579/4301/83593/7423/4303/5604/115727/9610/51378/5924/64926/7535/627/3480/2246/8517/382 |
| **hsa03018** | RNA degradation | 71 | -0,4160 | -1,5528 | 0,0116 | 0,0267 | 0,0123 | 4273 | tags=39%, list=23%, signal=31% | 54512/22894/29883/2023/57472/5073/9125/131870/11157/1656/51690/10200/3313/11340/28960/27258/51010/23016/80349/115752/5393/25804/87178/23658/3329/23404/56915 |
| **hsa04920** | Adipocytokine signaling pathway | 66 | 0,4131 | 1,5420 | 0,0146 | 0,0320 | 0,0147 | 4531 | tags=41%, list=24%, signal=31% | 7133/53632/3953/3667/4792/181/9021/126129/5465/7124/6774/23305/10000/2180/6513/8660/51422/8517/6517/5565/51094/8717/5443/6256/6257/5970/2182 |
| **hsa04915** | Estrogen signaling pathway | 116 | 0,4120 | 1,7129 | 0,0020 | 0,0068 | 0,0032 | 3550 | tags=29%, list=19%, signal=24% | 53358/3866/3762/3725/3868/2775/8689/114/6714/2100/4318/3763/196883/3710/1839/3304/3310/3872/2550/8202/112/3303/9586/25759/3708/115/10000/5914/5604/113/1509/6667/3860/111 |
| **hsa04360** | Axon guidance | 170 | 0,4098 | 1,7798 | 0,0020 | 0,0068 | 0,0032 | 2812 | tags=26%, list=15%, signal=22% | 4233/7474/10512/1946/25791/6714/4776/2047/90249/81029/5361/23365/8440/5747/22885/3897/8482/2049/55558/5727/659/54434/2043/4756/10501/2534/23380/817/84448/57522/57556/91584/54437/3611/5921/29984/7869/5533/219699/10509/3984/3688/10154/7220 |
| **hsa05146** | Amoebiasis | 97 | 0,4077 | 1,6426 | 0,0020 | 0,0068 | 0,0032 | 3267 | tags=32%, list=17%, signal=27% | 3909/3914/3554/2920/3576/1284/2921/3553/384/3586/22798/3908/7850/2335/3915/5747/5582/3684/909/1286/7124/911/7043/1437/3918/383/5579/735/7097/2774/9630 |
| **hsa04137** | Mitophagy - animal | 63 | 0,4075 | 1,5053 | 0,0187 | 0,0382 | 0,0176 | 2485 | tags=27%, list=13%, signal=24% | 3725/6714/3091/4286/79735/22808/10133/8878/22800/65018/665/7030/79065/664/598/23710/7942 |
| **hsa00190** | Oxidative phosphorylation | 115 | -0,4051 | -1,6366 | 0,0040 | 0,0119 | 0,0055 | 5173 | tags=54%, list=27%, signal=39% | 51079/1337/4541/4512/4728/6392/4719/4704/4513/4519/4723/4731/1537/6391/4716/55967/54539/29796/90423/4725/4713/6390/4722/4537/4697/7388/4717/10975/4538/245973/4702/4540/4696/4711/1329/1349/9377/4709/4701/126328/4715/1345/4714/4712/4707/4698/4706/4726/4514/4536/4535/27068/4508/4539/4718/1353/27089/4509/5464/56901/155066 |
| **hsa00310** | Lysine degradation | 58 | -0,4047 | -1,4601 | 0,0175 | 0,0363 | 0,0167 | 2591 | tags=28%, list=14%, signal=24% | 39/217/2146/1962/64754/51166/1892/6839/38/2639/3033/6419/79723/219/501 |
| **hsa05213** | Endometrial cancer | 57 | 0,4041 | 1,4690 | 0,0247 | 0,0474 | 0,0219 | 3328 | tags=32%, list=18%, signal=26% | 1026/10912/51176/6932/83439/999/673/1950/595/324/8312/3611/10297/10000/1495/5604/2309/1499 |
| **hsa04072** | Phospholipase D signaling pathway | 144 | 0,4009 | 1,6936 | 0,0021 | 0,0068 | 0,0032 | 3611 | tags=33%, list=19%, signal=27% | 53358/5154/8613/3576/114/56895/4254/5159/8877/9846/196883/23533/22808/27128/11069/10161/8612/2534/22800/8605/2918/5338/23396/5900/10672/9267/112/1950/2912/25759/5898/115/5337/7248/2768/10000/5155/9170/5604/160851/113/9162/26052/146850/382/111/5335 |
| **hsa04550** | Signaling pathways regulating pluripotency of stem cells | 131 | 0,4009 | 1,6793 | 0,0021 | 0,0068 | 0,0032 | 3950 | tags=35%, list=21%, signal=28% | 7474/2247/3976/130399/10637/7477/5603/90/463/7703/81029/8322/7482/6932/659/7483/1857/7476/84759/4088/80326/6498/324/3720/8312/10336/6774/3572/10297/657/10000/1855/4093/91/100532731/5604/1499/3480/652/93/4087/9314/1856/8325/7855/6926 |
| **hsa04514** | Cell adhesion molecules (CAMs) | 131 | 0,4004 | 1,6772 | 0,0021 | 0,0068 | 0,0032 | 3202 | tags=31%, list=17%, signal=26% | 4897/3696/5133/137075/923/940/6383/9080/1493/914/925/3685/6382/3897/7412/926/23562/3684/58494/29126/999/4756/29851/201633/80380/965/3133/10666/3117/24146/3134/1001/23308/80381/94030/3688/9074/23705/4684/958/3119 |
| **hsa04062** | Chemokine signaling pathway | 180 | 0,4002 | 1,7503 | 0,0020 | 0,0068 | 0,0032 | 3550 | tags=32%, list=19%, signal=26% | 6354/53358/2791/643/6364/2920/3576/6349/2921/114/2829/6714/10663/58191/7074/6375/2833/1230/6348/5747/196883/4792/23533/9564/6347/1236/1232/2826/6846/6369/1233/6362/673/112/3702/2268/2785/10803/6361/25759/6774/115/6352/10000/57580/54331/5579/409/5604/9560/113/1398/2309/146850/1234/8517/10451/111 |
| **hsa01522** | Endocrine resistance | 94 | 0,3951 | 1,5903 | 0,0060 | 0,0163 | 0,0075 | 3550 | tags=32%, list=19%, signal=26% | 53358/4854/28514/3725/1564/114/5603/6714/2100/3714/4318/1026/5747/196883/1839/1565/673/8202/112/4851/595/25759/115/10000/5604/113/4853/3480/6667/111 |
| **hsa04926** | Relaxin signaling pathway | 124 | 0,3926 | 1,6384 | 0,0021 | 0,0068 | 0,0032 | 3156 | tags=26%, list=17%, signal=22% | 4312/53358/2791/3725/7422/2775/2277/1284/114/5603/6714/7424/4318/196883/4792/1286/112/2785/9586/25759/115/10000/4842/1906/54331/1910/7423/409/5604/7046/7048/113 |
| **hsa04630** | JAK-STAT signaling pathway | 142 | 0,3921 | 1,6557 | 0,0021 | 0,0068 | 0,0032 | 2650 | tags=26%, list=14%, signal=23% | 5154/3976/29949/11009/163702/3563/5159/3586/9180/1026/3953/3560/6775/316/9021/1438/9655/1387/3566/2670/3596/9306/5292/1950/595/3459/4170/6774/3572/598/1437/10000/3600/5155/2057/3575/3588 |
| **hsa04657** | IL-17 signaling pathway | 85 | 0,3909 | 1,5301 | 0,0104 | 0,0242 | 0,0112 | 2140 | tags=21%, list=11%, signal=19% | 4312/6354/3725/6364/2920/3576/2921/1051/3553/5603/4318/7128/4792/6347/23118/3596/7124/6361 |
| **hsa04974** | Protein digestion and absorption | 85 | 0,3903 | 1,5278 | 0,0104 | 0,0242 | 0,0112 | 2980 | tags=29%, list=16%, signal=25% | 4224/1294/4311/117247/1284/1306/255631/4225/6550/340024/1292/1291/1803/2006/256076/9056/1286/23428/486/206358/3783/5644/6543/6520/482 |
| **hsa04726** | Serotonergic synapse | 102 | 0,3901 | 1,5849 | 0,0102 | 0,0242 | 0,0112 | 3148 | tags=25%, list=17%, signal=21% | 2791/3762/2775/1564/1843/3763/3710/5582/1565/3363/239/8605/778/673/3357/351/2785/3708/54331/5579/7220/5743/774/5604/4128 |
| **hsa04012** | ErbB signaling pathway | 81 | 0,3891 | 1,5127 | 0,0161 | 0,0342 | 0,0158 | 4441 | tags=35%, list=23%, signal=27% | 53358/3725/2069/374/6714/1026/8440/5747/1839/5582/2065/817/673/1950/868/25759/10000/5579/5604/867/1398/5335/1399/5609/816/815/6654/27 |
| **hsa04713** | Circadian entrainment | 84 | 0,3869 | 1,5071 | 0,0166 | 0,0349 | 0,0161 | 4281 | tags=36%, list=23%, signal=28% | 2791/3762/2977/2775/114/5187/3763/196883/51655/3710/5582/6262/817/112/2785/2904/3708/115/4842/54331/55811/5579/8913/113/111/8911/8863/816/815/5331 |
| **hsa04371** | Apelin signaling pathway | 129 | 0,3865 | 1,6233 | 0,0020 | 0,0068 | 0,0032 | 3592 | tags=32%, list=19%, signal=26% | 6696/2791/4854/114/5054/8877/10365/53632/814/196883/23533/4635/22808/3710/6262/999/5327/22800/4088/10672/112/595/2785/3708/100271849/115/23710/10000/4842/54331/6543/55811/5604/7046/113/51422/146850/10014/4087/111/5581 |
| **hsa05152** | Tuberculosis | 161 | 0,3864 | 1,6633 | 0,0020 | 0,0068 | 0,0032 | 4502 | tags=35%, list=24%, signal=27% | 54106/3687/3656/1051/3553/2212/5603/6714/8877/3586/3552/245972/23365/10333/1379/10892/3684/4360/1387/817/535/7124/10312/3459/537/7421/3117/1263/5533/7043/2209/10000/3588/8767/7097/820/3119/2213/3916/26253/1509/718/2033/51606/4261/4046/8717/3587/2207/3460/816/7189/815/8844/5970/30835 |
| **hsa04350** | TGF-beta signaling pathway | 88 | 0,3821 | 1,5067 | 0,0186 | 0,0382 | 0,0176 | 4060 | tags=39%, list=21%, signal=31% | 130399/10637/90/9765/285704/659/4092/1387/4756/8646/4088/7124/57817/64388/9241/7043/657/9372/4093/5516/7050/91/7046/7057/7048/2033/6667/652/4091/93/4087/1030/1875/1634 |
| **hsa01521** | EGFR tyrosine kinase inhibitor resistance | 78 | 0,3817 | 1,4709 | 0,0207 | 0,0415 | 0,0191 | 3611 | tags=27%, list=19%, signal=22% | 4233/53358/5154/2247/7422/6714/5159/5582/2065/673/1950/25759/6774/598/10000/5155/5579/5604/2309/3480/5335 |
| **hsa05231** | Choline metabolism in cancer | 94 | 0,3791 | 1,5261 | 0,0160 | 0,0342 | 0,0158 | 3611 | tags=29%, list=19%, signal=23% | 5154/3725/10810/8613/5159/3091/6580/5582/8612/8605/5338/23396/5900/1950/1120/5337/7248/10000/5155/5579/5604/160851/9162/6581/6667/56261/5335 |
| **hsa04750** | Inflammatory mediator regulation of TRP channels | 93 | 0,3786 | 1,5206 | 0,0161 | 0,0342 | 0,0158 | 4516 | tags=37%, list=24%, signal=28% | 3554/114/3553/5603/6714/196883/3710/5582/817/239/8605/112/3357/79054/3708/115/51802/8398/5579/9311/113/41/111/162514/5581/5335/5583/3269/3356/816/815/5331/5732/51393 |
| **hsa04650** | Natural killer cell mediated cytotoxicity | 112 | 0,3775 | 1,5550 | 0,0101 | 0,0242 | 0,0112 | 5724 | tags=45%, list=30%, signal=31% | 53358/9437/3002/27040/9436/356/117157/919/3932/5582/2534/3821/673/3805/7124/3459/3133/25759/5551/5533/1437/5579/962/5604/3811/6452/7535/22914/10451/5335/2207/3824/3460/3106/3937/6654/3383/5777/3135/4773/7305/3809/3812/100507436/4772/3804/3455/5894/7409/3802 |
| **hsa05224** | Breast cancer | 136 | 0,3769 | 1,5922 | 0,0021 | 0,0068 | 0,0032 | 2663 | tags=26%, list=14%, signal=23% | 7474/53358/2247/4854/28514/3725/3280/7477/2100/3714/1026/81029/8322/10912/2254/51176/4791/7482/6932/7483/83439/1857/8822/7476/673/8202/80326/4851/1950/595/324/8312/25759/10297/10000/1855 |
| **hsa05202** | Transcriptional misregulation in cancer | 157 | 0,3764 | 1,6215 | 0,0020 | 0,0068 | 0,0032 | 4131 | tags=36%, list=22%, signal=29% | 4233/8091/5154/9915/604/8013/3576/1051/5468/3002/7849/597/4318/1026/4286/5328/1848/7850/10912/5747/3560/64919/7185/3684/7709/5327/64332/1436/1668/5087/5090/2308/7030/2321/7102/598/860/2209/1437/5914/100532731/26471/3486/7048/958/330/3480/6667/1649/4094/51426/6688/221037/6256/472/51274/6257 |
| **hsa04724** | Glutamatergic synapse | 106 | 0,3759 | 1,5330 | 0,0102 | 0,0242 | 0,0112 | 2812 | tags=25%, list=15%, signal=22% | 6506/2791/2775/50944/114/1742/9229/196883/3710/5582/8605/2918/5338/2752/112/2912/2785/2904/3708/5533/115/5337/27165/116444/54331/5579/7220 |
| **hsa04625** | C-type lectin receptor signaling pathway | 101 | 0,3754 | 1,5232 | 0,0122 | 0,0274 | 0,0126 | 4502 | tags=35%, list=24%, signal=27% | 3725/1959/3553/5603/6714/4776/3586/23365/4792/4791/22808/3710/10892/22800/7124/602/868/6361/1263/3708/5533/10000/5971/5743/3558/26253/8517/9020/10379/4046/2207/9261/8844/5970/30835 |
| **hsa04540** | Gap junction | 80 | 0,3748 | 1,4523 | 0,0224 | 0,0444 | 0,0205 | 3156 | tags=28%, list=17%, signal=23% | 5154/2977/114/6714/5159/10381/196883/3710/5582/112/3357/1950/3708/115/7082/1453/5155/7280/5579/10746/5604/113 |
| **hsa04935** | Growth hormone synthesis, secretion and action | 109 | 0,3737 | 1,5321 | 0,0102 | 0,0242 | 0,0112 | 3785 | tags=30%, list=20%, signal=24% | 53358/114/5603/3667/5747/196883/3483/9564/3710/5582/9021/1387/3726/778/112/9586/25759/6774/3708/115/10000/55811/5579/3486/5604/113/1398/8660/2033/111/5335/1399/6753 |
| **hsa04390** | Hippo signaling pathway | 144 | 0,3735 | 1,5779 | 0,0021 | 0,0068 | 0,0032 | 4017 | tags=39%, list=21%, signal=31% | 7474/7477/374/5054/27113/1742/81029/8322/51176/7482/6932/659/7483/83439/1857/4092/999/7476/4088/80326/23418/595/324/7159/166824/126374/8312/1740/84962/122786/7043/10297/1741/657/1453/10207/1454/1855/329/25937/5516/1495/7046/7048/6788/1499/55844/2246/652/5521/4087/3993/1856/8325/7855/8994 |
| **hsa05130** | Pathogenic Escherichia coli infection | 185 | 0,3731 | 1,6295 | 0,0020 | 0,0068 | 0,0032 | 2946 | tags=24%, list=16%, signal=20% | 2017/3725/10810/3554/10787/3576/137075/3553/2212/5603/356/10381/147945/9080/9826/4643/10458/23365/8440/4792/4641/7100/27128/23562/4646/23118/4644/2534/7456/10672/9267/7124/8976/4645/24146/7082/4642/2768/22989/3688/9074/7280/9170/57644 |
| **hsa04912** | GnRH signaling pathway | 87 | 0,3729 | 1,4743 | 0,0224 | 0,0444 | 0,0205 | 4430 | tags=33%, list=23%, signal=26% | 3725/114/5603/6714/196883/4323/3710/1839/817/8605/5338/778/2796/112/3708/115/5337/5579/2797/10746/5604/113/111/5609/816/815/5331/6654/4215 |
| **hsa04142** | Lysosome | 122 | 0,3674 | 1,5345 | 0,0081 | 0,0204 | 0,0094 | 4243 | tags=36%, list=22%, signal=28% | 1514/6556/22901/1512/4864/3482/245972/1508/6448/54/5660/427/3074/5641/1513/4126/535/4758/4891/10312/537/1201/27074/3988/3423/57192/3373/84572/1515/26503/9516/3916/1509/51606/2799/2588/967/950/9583/10577/1519/8905/1497/3073 |
| **hsa04140** | Autophagy - animal | 132 | 0,3625 | 1,5220 | 0,0042 | 0,0121 | 0,0056 | 6253 | tags=49%, list=33%, signal=33% | 1514/54541/55062/3091/1612/1508/3667/22808/8878/22800/3476/8897/79065/664/3708/598/7248/23710/53349/10000/22863/5516/140775/203228/83452/23604/5604/8660/10325/3916/1509/81671/3480/2081/10010/1613/89849/8408/7189/23130/23192/51100/84971/64121/116442/6237/11345/5728/5894/11337/338382/57521/7249/58476/8837/29110/5605/6794/115201/9821/5170/7405/84335/5861/10670 |
| **hsa05200** | Pathways in cancer | 493 | 0,3583 | 1,7265 | 0,0022 | 0,0068 | 0,0032 | 4248 | tags=36%, list=22%, signal=29% | 4233/4312/7474/2791/3909/5154/2247/4854/28514/3725/9915/3914/7422/2737/2277/3280/7477/3576/1284/114/5468/2100/3563/7849/4254/5159/356/7424/3091/27113/238/3714/22798/3675/4318/3908/1026/4286/9826/2034/1612/81029/2335/3915/8322/10912/2254/23365/5979/5747/3685/196883/51176/4792/3560/4791/7185/5727/6775/7482/5467/6932/5582/7483/83439/10125/1857/8822/1438/10161/7709/999/119391/7476/1436/1387/817/3566/5338/4088/5900/1286/10672/673/8202/2308/80326/5228/3596/112/4851/5292/1950/595/324/3459/2785/54583/8312/7296/2113/2949/5898/25780/6774/3572/115/5337/598/7043/10297/2768/10000/3918/64399/3600/5155/3688/2057/1906/3575/1855/3673/54331/329/5579/5914/9170/6513/5743/1910/1495/83593/7423/23604/5604/7046/867/7048/113/115727/1398/3558/4853/1499/330/2033/3480/6667/2246/8517/652/4087/111/4780/5335/1399/1030/51426/1439/6688/1613/6256/1856/8325/7855/3913/3912/7428/9446/6257/284217/3460/2253/816/7852/7189/815/388585 |
| **hsa05165** | Human papillomavirus infection | 314 | 0,3565 | 1,6585 | 0,0021 | 0,0068 | 0,0032 | 5011 | tags=41%, list=26%, signal=31% | 7474/6696/3696/3909/4854/3914/3690/7422/3280/534/7477/1284/22801/5159/356/22798/245972/3675/3908/1026/81029/2335/3915/8322/84441/3672/5747/3685/1292/1291/5986/5526/7482/6932/256076/7483/83439/1857/9296/7476/1387/57801/535/7143/7148/1286/2308/80326/7450/4851/7124/1950/595/324/10312/5649/9586/537/3133/8312/1740/3134/8638/7248/10297/1741/10000/3918/10207/3688/3694/1855/3673/148022/5516/7060/5743/3693/5604/7057/9223/4853/1499/2033/55844/51606/8517/5521/23352/1280/8516/10379/3993/5525/8717/1856/8325/7855/3913/3912/472/1293/284217/525/526/9794/3106/388585/528/6654/9550/7297/3516/5970/131873/523/527/7058/5829/50617/1101/2932/3135/1278/5527/55502/92359/3551 |
| **hsa04810** | Regulation of actin cytoskeleton | 197 | 0,3564 | 1,5702 | 0,0021 | 0,0068 | 0,0032 | 3481 | tags=26%, list=18%, signal=21% | 3696/5154/2247/1131/3690/3687/10787/85477/6714/22801/5159/7074/3675/2335/10458/2254/23365/3672/5747/3685/9564/22808/54434/3684/8822/22800/23396/10672/673/1950/324/8976/3681/8826/10297/2768/3984/5155/3688/3694/3673/89846/9170/5962/3693/5604/1398/2934/2246/10451/54776 |
| **hsa04024** | cAMP signaling pathway | 186 | 0,3563 | 1,5520 | 0,0021 | 0,0068 | 0,0032 | 3550 | tags=28%, list=19%, signal=23% | 5141/3725/2737/114/7074/135/27198/814/610/5139/196883/4792/5727/2740/11069/6262/1387/5465/5348/5020/817/338442/22800/2550/5338/5143/778/673/8843/112/486/9586/7434/2904/115/5337/116444/64411/10000/64399/1906/55811/4301/4886/482/5604/7137/113/2033/627/10451/111 |
| **hsa04066** | HIF-1 signaling pathway | 104 | 0,3558 | 1,4460 | 0,0245 | 0,0472 | 0,0218 | 4676 | tags=38%, list=25%, signal=29% | 7422/2026/5054/3091/5209/1026/5582/7018/1387/817/1950/2321/92483/3459/54583/3099/8569/6774/10000/1906/5579/6513/7076/5604/229/51378/2033/5214/3480/5335/7428/3460/816/815/7010/5970/3162/2027/2872/3098 |
| **hsa05206** | MicroRNAs in cancer | 200 | 0,3539 | 1,5605 | 0,0021 | 0,0068 | 0,0032 | 3714 | tags=32%, list=20%, signal=26% | 4233/8091/5154/4854/3690/7422/10253/406992/54541/23414/1946/407018/5159/4318/1026/7431/5328/3667/7168/659/5582/8626/2065/960/5243/4082/7078/1387/407010/7143/7148/1545/4851/5292/595/324/407031/442898/4170/6774/406881/27165/6768/10297/406913/5155/5579/100532731/5962/5743/5604/7057/1398/8660/4853/2033/599/10014/23405/5581/5335/407011/1399 |
| **hsa04151** | PI3K-Akt signaling pathway | 320 | 0,3510 | 1,6291 | 0,0022 | 0,0068 | 0,0032 | 3538 | tags=29%, list=19%, signal=24% | 4233/6696/2791/3696/3909/5154/2247/2069/3914/3690/7422/2277/374/1284/54541/1946/22801/3563/4254/5159/356/7424/9180/22798/3675/3908/1026/6446/2335/3915/1435/3667/2254/3672/5747/3685/1292/1291/23533/3560/5526/256076/8822/10161/2065/1436/3566/7143/7148/1286/3164/5228/7450/1950/595/2321/2785/5649/9586/200186/4170/598/7248/10000/3918/5155/3688/2057/3575/3694/3673/54331/5516/9170/7060/3693/10110/7423/7097/5604/7057/9223/3558/51378/2309/146850/55844/627/3480/2246/8517/5521/1280 |
| **hsa05132** | Salmonella infection | 205 | 0,3484 | 1,5407 | 0,0021 | 0,0068 | 0,0032 | 5666 | tags=40%, list=30%, signal=28% | 3725/10810/54106/10787/388/3576/2318/3553/5603/51429/23207/51176/4792/7100/10333/113146/6932/27128/83439/257364/399/4646/79026/23118/147700/29109/9267/7124/8976/23643/5898/6281/22989/2316/112574/10000/329/8767/57644/9842/7097/5604/1499/330/8517/89953/382/55823/837/10006/8717/302/5609/7189/23191/1785/5970/83547/6993/1783/5869/121512/9367/197259/9266/3551/4628/5788/58484/10398/4627/1778/2317/6237/114548/64837/257397/5894/4790/338382/23339 |
| **hsa04921** | Oxytocin signaling pathway | 144 | 0,3444 | 1,4547 | 0,0106 | 0,0246 | 0,0113 | 2372 | tags=21%, list=12%, signal=18% | 59283/3762/3725/2977/2775/114/9254/6714/4776/57172/1026/53632/814/3763/196883/23533/3710/5582/6262/4882/5020/817/8605/59285/778/112/595/3708/5533/115 |
| **hsa05016** | Huntington disease | 177 | -0,3434 | -1,4606 | 8,0000 | 0,0204 | 0,0094 | 5216 | tags=44%, list=27%, signal=32% | 292/51079/1337/83544/4512/4728/3065/7416/6392/4719/4704/4513/6647/4519/4723/4731/1537/6391/5441/4716/55967/54539/5432/29796/5437/4725/1212/581/4713/6390/4722/4697/7388/7802/4717/10975/4702/5436/4696/4711/1329/1349/9377/4709/4701/126328/5440/4715/8701/1345/4714/1768/4712/4707/4698/4706/4726/4514/7419/3066/8218/4508/7019/4718/55081/5438/27089/7157/4509/54205/56901/5433/6875/148327/2902/2882 |
| **hsa05226** | Gastric cancer | 135 | 0,3398 | 1,4305 | 0,0169 | 0,0354 | 0,0163 | 3949 | tags=33%, list=21%, signal=27% | 4233/7474/53358/2247/7477/1026/81029/8322/10912/2254/51176/7482/6932/7483/83439/1857/8822/999/7476/5243/4088/673/80326/1950/595/324/8312/25759/7043/10297/10000/1855/1495/5604/7046/7048/1499/2246/4087/1030/51426/6256/1856/8325/7855 |
| **hsa04145** | Phagosome | 144 | 0,3351 | 1,4156 | 0,0127 | 0,0284 | 0,0131 | 4872 | tags=40%, list=26%, signal=30% | 1514/3690/78989/534/2212/4688/10381/245972/2204/3685/10333/3684/4360/9296/4481/535/81035/715/10312/537/3133/9146/3117/3134/2209/4973/3688/4842/3673/7280/7060/729238/3693/7097/7057/3119/2213/3916/718/51606/8417/525/526/3106/528/9550/30835/83547/81027/523/9341/55176/527/7058/1783/50617/5869/3135 |
| **hsa04270** | Vascular smooth muscle contraction | 118 | 0,3350 | 1,3955 | 0,0240 | 0,0470 | 0,0217 | 3613 | tags=25%, list=19%, signal=21% | 146/2977/114/135/9826/23365/196883/3710/5582/4882/3778/8605/778/10672/673/112/133/5739/3708/115/2768/1906/8398/5579/5604/113/54776/111/5581/5583 |
| **hsa04621** | NOD-like receptor signaling pathway | 157 | 0,3328 | 1,4337 | 0,0121 | 0,0273 | 0,0126 | 4516 | tags=31%, list=24%, signal=24% | 3725/2920/3576/5027/2921/3553/199713/5603/22861/1508/7128/4792/6347/3710/23118/10135/1668/3665/388646/7124/3708/598/6352/23710/24145/148022/329/8767/1669/22900/820/10628/55914/171389/330/8517/837/2634/10010/10379/118429/3428/2635/7189/5331/7297/4938/5970/51393 |
| **hsa05167** | Kaposi sarcoma-associated herpesvirus infection | 171 | 0,3321 | 1,4417 | 0,0099 | 0,0242 | 0,0112 | 5628 | tags=43%, list=30%, signal=30% | 2791/2247/3725/7422/2920/3576/2921/5603/6714/4776/3091/1026/1230/4792/23533/3710/1232/1387/3665/1233/595/3459/2785/3133/6774/3708/5533/3134/3572/23710/1437/10000/5155/57580/148022/54331/22863/5743/5604/146850/718/1499/1234/2033/8517/5335/10379/8717/5609/3106/9261/7297/5970/3383/2932/3135/4773/7538/4067/3551/3716/5925/2919/1237/100507436/94235/4772/3455/2790/11345/5894/4790/11337 |
| **hsa04714** | Thermogenesis | 209 | -0,3304 | -1,4394 | 0,0077 | 0,0199 | 0,0092 | 4906 | tags=42%, list=26%, signal=32% | 23028/4728/6392/4719/4704/4513/5562/6198/4519/51703/5567/4723/4731/1537/6391/4716/55967/54539/29796/4725/5600/3991/4713/6597/6390/6199/4722/4537/4697/109/7388/4717/10975/4538/1374/4702/6605/4540/788/4696/4711/1329/51241/5593/1349/9377/4709/4701/126328/25915/4715/1345/4714/60/4712/64223/4707/6300/51103/4698/6599/4706/65260/4726/4514/4536/152831/4535/1376/91942/8193/4508/4539/285521/4718/2260/29078/1353/28958/137682/51287/27089/4509/107/56901/86/148327 |
| **hsa04068** | FoxO signaling pathway | 125 | 0,3297 | 1,3811 | 0,0243 | 0,0472 | 0,0218 | 3755 | tags=33%, list=20%, signal=26% | 604/5603/356/10365/3586/1026/6446/53632/114907/10912/3667/1387/4088/673/2308/1950/595/664/6774/1263/7043/23710/10000/3575/1454/10110/901/4303/5604/7046/7048/8660/2309/51422/2033/3480/6648/51701/6517/5565/1030 |
| **hsa05163** | Human cytomegalovirus infection | 206 | 0,3293 | 1,4594 | 0,0063 | 0,0163 | 0,0075 | 3714 | tags=28%, list=20%, signal=23% | 2791/3690/7422/2775/3554/3576/6349/114/3553/5603/6714/4776/356/1026/9826/1230/6348/23365/5747/3685/196883/4792/9564/6347/3710/5582/1232/10672/112/7124/595/2785/9586/3133/6774/3708/5533/3134/115/6352/7248/2768/10000/3588/54331/5579/5743/5604/9560/113/1398/1499/1234/6667/8517/111/11214/1399 |
| **hsa04144** | Endocytosis | 244 | 0,3277 | 1,4771 | 0,0021 | 0,0068 | 0,0032 | 5058 | tags=35%, list=27%, signal=26% | 116988/23362/6714/23327/138429/1601/3482/9765/50807/155382/274/9922/3560/27128/3304/55048/857/3310/84440/92421/5338/23396/4088/7456/22905/3798/9267/3303/116985/868/8976/116987/3133/9146/84249/642517/3134/5662/5337/64411/9372/100287171/26056/729092/409/7046/867/7048/26052/60682/1234/100526767/3480/10890/382/4087/79720/116984/3305/6455/118813/161/2348/7852/3106/7189/57403/1785/57132/51510/414189/253725/11031/147179/83737/9230/64145/119016/51100/5869/64744/9266/3135/57154/858/6643 |
| **hsa05131** | Shigellosis | 200 | 0,3148 | 1,3884 | 0,0146 | 0,0320 | 0,0147 | 5871 | tags=39%, list=31%, signal=27% | 2017/3725/3554/3576/3553/55062/5603/6714/824/5747/4792/7100/3710/10892/960/8878/23118/2706/2308/7124/8976/3099/199746/664/3611/3708/598/6352/23710/831/1437/10000/3688/22863/8767/4303/1398/10325/2309/718/8517/5581/837/5335/1399/8717/2931/472/7189/5331/5970/7335/10318/3098/5829/2932/7094/4636/3551/23291/58484/10398/64121/10163/5332/3678/114548/257397/63916/11345/4790/11337/57521/5217/29110/10627/113026/8915 |
