# Supplementary material for: 5-aminoimidazole-4-carboxamide ribonucleoside induces differentiation in a subset of primary acute myeloid leukemia blasts
Source: BMC Cancer. 2020 Nov 11;20:1090. doi: 10.1186/s12885-020-07533-6 (PMC7657321; doi:10.1186/s12885-020-07533-6)
Supplement: Supplementary file 4 — Additional file 4 Supplementary Table 4. Common gene expression changes of control and AICAr-treated bone marrow sample (Pt14) and U937 cells in relation to their respective untreated controls (selected from the results of the differential gene expression analysis conducted by GENEWIZ). [file 12885_2020_7533_MOESM4_ESM.docx]

**Supplementary Table 4**. **Common gene expression changes of control and AICAr-treated bone marrow sample (Pt14) and U937 cells in relation to their respective untreated controls**

| GENES UPREGULATED | GENES DOWNREGULATED |
| --- | --- |
| \| ABHD4 \| \| --- \| \| BATF2 \| \| C6orf223 \| \| CCL2 \| \| CCL3 \| \| CCR1 \| \| CDCP1 \| \| CDKN1A \| \| CRISPLD2 \| \| DCSTAMP \| \| DLEU7 \| \| EFCAB12 \| \| GPR65 \| \| GPR68 \| \| GRAP2 \| \| GTF2IRD2 \| \| GVINP1 \| \| HELZ2 \| \| IL1B \| \| IL1RN \| \| IL4I1 \| \| IPP \| \| IRF7 \| \| LRRC63 \| \| NLRP1 \| \| OASL \| \| PCDHGC3 \| \| PTGS2 \| \| RASSF8 \| \| RNASE6 \| \| SERTAD1 \| \| SHISA4 \| \| SIGLEC15 \| \| SMIM25 \| \| TCF7 \| \| TDRD9 \| \| TLR6 \| \| TMEM150B \| \| TRAC \| \| VWA5A \| | \| AC009086.2 \| \| --- \| \| AC022413.1 \| \| AC083880.1 \| \| AC099568.2 \| \| AC110285.7 \| \| AC135050.1 \| \| ACTL10 \| \| AL135925.1 \| \| AL136162.1 \| \| AL354892.2 \| \| ANKRD63 \| \| AP003774.1 \| \| ASCL2 \| \| AVPR2 \| \| CITED1 \| \| FARSA-AS1 \| \| FBXL22 \| \| LINC00641 \| \| LINC01521 \| \| LNP1 \| \| MAN1B1-AS1 \| \| MAP6D1 \| \| MATN4 \| \| MYOM2 \| \| NEURL1 \| \| PANO1 \| \| PGBD4 \| \| PPP1R10 \| \| SNORD100 \| \| SNORD14A \| \| SPARC \| \| TSPOAP1 \| \| TUBA3D \| \| UFSP1 \| \| ZBTB12 \| |
