# Supplementary material for: 5-aminoimidazole-4-carboxamide ribonucleoside induces differentiation in a subset of primary acute myeloid leukemia blasts
Source: BMC Cancer. 2020 Nov 11;20:1090. doi: 10.1186/s12885-020-07533-6 (PMC7657321; doi:10.1186/s12885-020-07533-6)

Supplementary Figure 1

a)

TCGA Dataset, N = 162

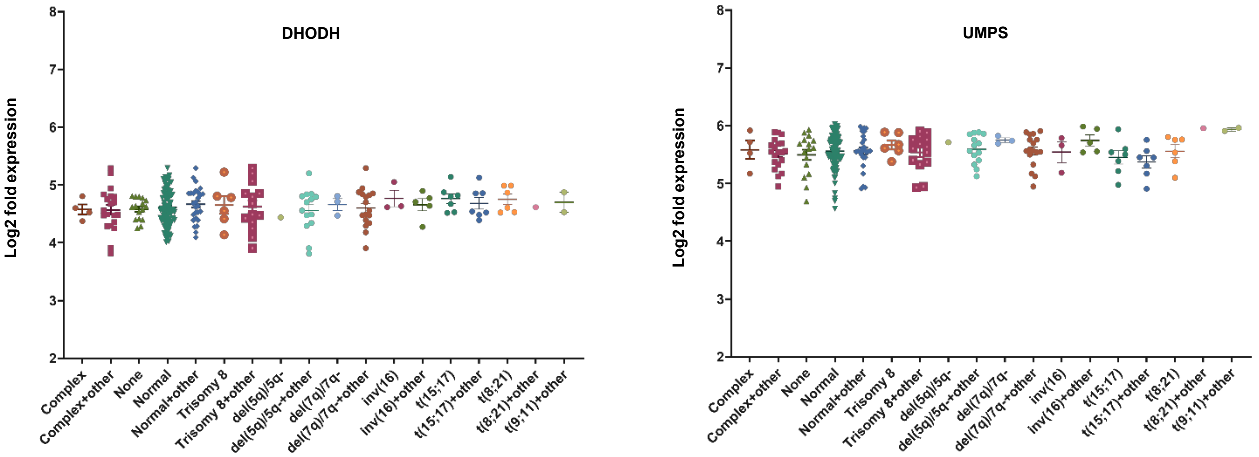

GSE15434 Dataset, Normal kariotype AML, N= 251

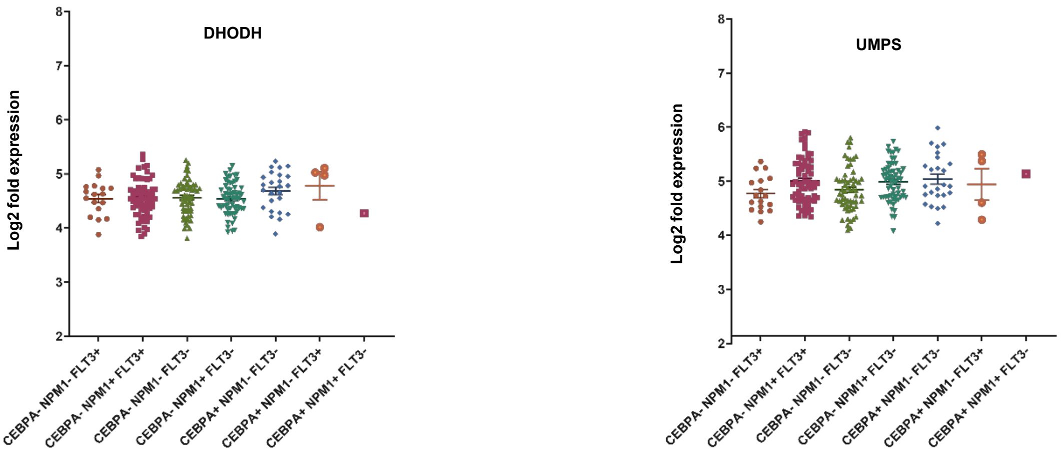

b)

TCGA Dataset, N = 162

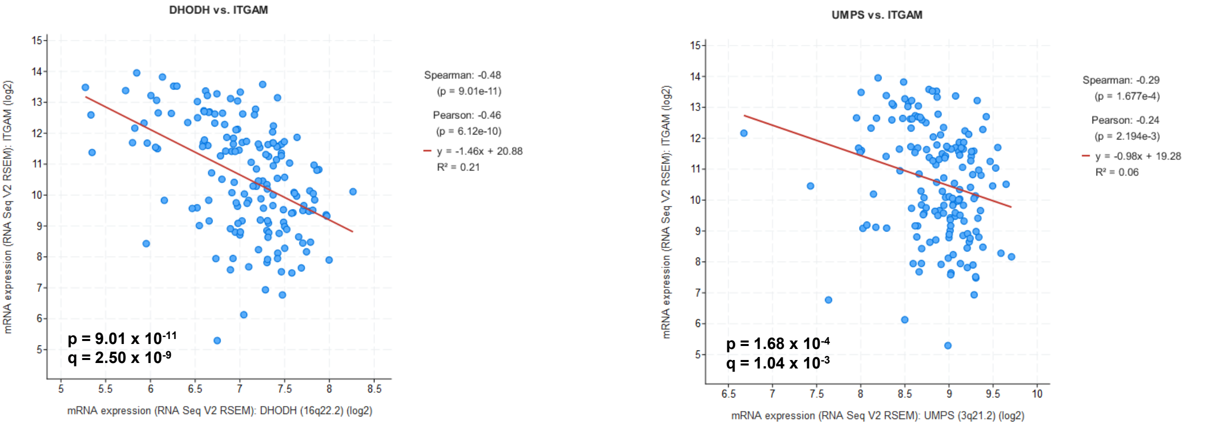

OHSU Dataset, N = 405

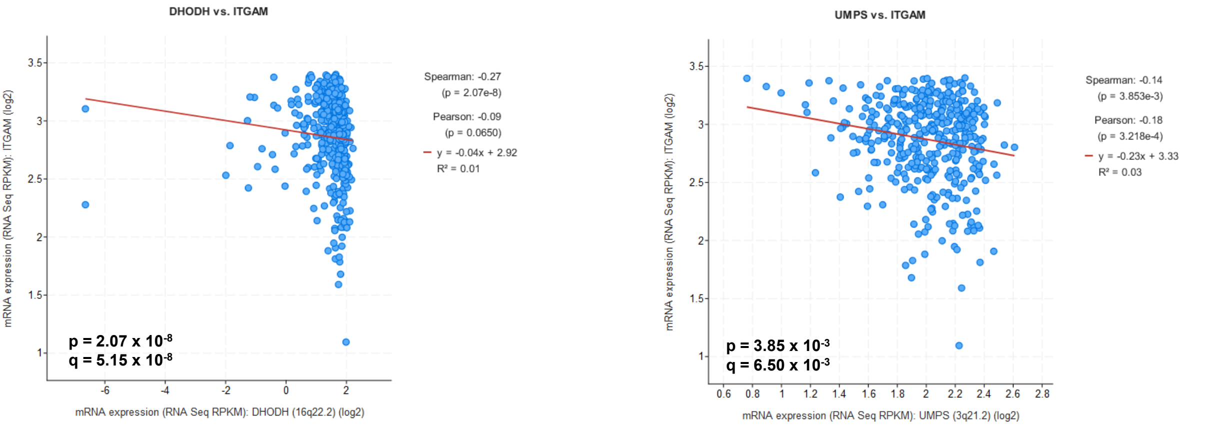

Supplement: Supplementary file 5 — Additional file 5 Supplementary Figure 1. Expression of DHODH and UMPS in AML patient samples. A) Publicly available datasets TCGA and GSE15434 (accessed through Bloodspot database) with a total of 413 patients were used to compare expression levels of DHODH and UMPS in primary AML samples in accordance to their cytogenetics and mutational status of CEBPA, FLT3 and NPM1. B) Gene co-expression analysis between DHODH or UMPS and ITGAM (CD11b) was performed on publicly available datasets TCGA and OHSU (accessed through cBioPortal database) which jointly comprise 567 samples. [file 12885_2020_7533_MOESM5_ESM.pdf]
